# Globalization, first-foods systems transformations and corporate power: a synthesis of literature and data on the market and political practices of the transnational baby food industry

**DOI:** 10.1186/s12992-021-00708-1

**Published:** 2021-05-21

**Authors:** Phillip Baker, Katheryn Russ, Manho Kang, Thiago M. Santos, Paulo A. R. Neves, Julie Smith, Gillian Kingston, Melissa Mialon, Mark Lawrence, Benjamin Wood, Rob Moodie, David Clark, Katherine Sievert, Monique Boatwright, David McCoy

**Affiliations:** 1 Institute for Physical Activity and Nutrition, Deakin University, Geelong, Australia; 2 University of California, Davis, CA USA; 3 International Centre for Equity in Health, Federal University of Pelotas, Pelotas, Brazil; 4 Research School of Population Health, Australian National University, Canberra, Australia; 5 Centre for Primary Care and Public Health, Queen Mary University, London, UK; 6 Trinity Business School, Trinity College Dublin, Dublin, Ireland; 7 School of Health and Social Development, Deakin University, Geelong, Australia; 8 Melbourne School of Population and Global Health, University of Melbourne, Melbourne, Australia; 9 Independent Consultant on Public Health Law, New York, USA; 10 School of Exercise and Nutrition Sciences, Deakin University, Geelong, Australia

**Keywords:** Infant formula, Milk formula, Breastmilk substitutes, Breastfeeding, Commercial determinants of health, Corporate power, Baby food industry, Lobbying, Corporate science, Food systems

## Abstract

**Background:**

The global milk formula market has ‘boomed’ in recent decades, raising serious concerns for breastfeeding, and child and maternal health. Despite these developments, few studies have investigated the global expansion of the baby food industry, nor the market and political practices corporations have used to grow and sustain their markets. In this paper, our aim is to understand the strategies used by the baby food industry to shape ‘first-foods systems’ across its diverse markets, and in doing so, drive milk formula consumption on a global scale. We used a theoretically guided synthesis review method, which integrated diverse qualitative and quantitative data sources.

**Results:**

Global milk formula sales grew from ~US$1.5 billion in 1978 to US$55.6 billion in 2019. This remarkable expansion has occurred along two main historical axes. First, the widening geographical reach of the baby food industry and its marketing practices, both globally and within countries, as corporations have pursued new growth opportunities, especially in the Global South. Second, the broadening of product ranges beyond infant formula, to include an array of follow-up, toddler and specialized formulas for a wider range of age groups and conditions, thereby widening the scope of mother-child populations subject to commodification. Sophisticated marketing techniques have been used to grow and sustain milk formula consumption, including marketing through health systems, mass-media and digital advertising, and novel product innovations backed by corporate science. To enable and sustain this marketing, the industry has engaged in diverse political practices to foster favourable policy, regulatory and knowledge environments. This has included lobbying international and national policy-makers, generating and deploying favourable science, leveraging global trade rules and adopting corporate policies to counter regulatory action by governments.

**Conclusion:**

The baby food industry uses integrated market and political strategies to shape first-foods systems in ways that drive and sustain milk formula market expansion, on a global scale. Such practices are a major impediment to global implementation of the International Code of Marketing of Breastmilk Substitutes, and other policy actions to protect, promote and support breastfeeding. New modalities of public health action are needed to negate the political practices of the industry in particular, and ultimately to constrain corporate power over the mother-child breastfeeding dyad.

**Supplementary Information:**

The online version contains supplementary material available at 10.1186/s12992-021-00708-1.

## Background

The commercial determinants of health (CDOH) are receiving growing attention from researchers, advocates and policy-makers, with the purpose of informing societal responses to so-called ‘manufactured’ or ‘industrial’ epidemics, and the need to address corporate power as an urgent public health priority [1–4]. In this paper, we focus on the commercial determinants of maternal, newborn and child health. Our aim is to understand the power of the transnational baby food industry to shape ‘first-foods systems’ in ways that drive milk formula consumption, and in doing so, undermine breastfeeding on a global scale.

The mother-child breastfeeding dyad is a powerful force for sustainable development. As the biological ‘first-food’ for human children, breastmilk is safe to consume, nutritionally optimised to the child’s evolving developmental needs, and protects against infection [5, 6]. It is literally ‘packaged with love’ given breastfeeding fosters mother-child bonding, and reduces stress for both [7]. The breastfed child is more likely to achieve their full intellectual potential, and hence perform better at school and work in later life [8]. Near universal breastfeeding would save an estimated 823,000 deaths in children under-5 years of age, and 98,000 maternal deaths from cancer and type-2 diabetes every year [8, 9]. For children, not breastfeeding increases the risk of all-cause mortality, diarrhoea, respiratory infection and dental malocclusion, and likely obesity and type-2 diabetes, and for mothers the risk of breast cancer, and likely ovarian cancer and type-2 diabetes [8]. To ensure child survival, optimal development and health, the World Health Organization (WHO) recommends infants initiate breastfeeding in the first hour of life, are then exclusively breastfed for 6 months, and thereafter receive nutritious and safe complementary foods, while breastfeeding continues for up to 2 years of age or beyond [10].

Yet according to UNICEF’s latest estimates, just 49% of newborns initiate breastfeeding within the first hour of life, 44% are exclusively breastfed to 6 months, and 44% continue to breastfeed at 2 years of age [11, 12]. One key explanation for these low global breastfeeding rates, is the aggressive marketing and promotion of breastmilk substitutes (BMS). Exposure to such marketing results in reduced breastfeeding initiation, exclusivity and duration, irrespective of country context [13–15]. Only a small proportion of mothers are unable to breastfeed for physiological or medical reasons, yet many more do not because they are the denied the choice, or lack the support to do so. For these reasons, BMS are made available as regulated food products [16, 17]. Milk formulas are the main type of BMS consumed worldwide, defined as foods marketed or otherwise represented as partial or total replacements for breastmilk, including any milk drinks marketed for ages 0–36 months [18]. Categories include standard infant formula (0–6 months), follow-up formula (7–12 months), growing-up (or toddler) milks (13–36 months) and specialised formulas. By definition, milk formulas are ultra-processed foods [19, 20], typically formulations of powdered milk proteins, vegetable oils, lactose and other sugars, micronutrients and cosmetic additives [21–23].

Milk formulas are implicated in child malnutrition through the displacement of breastfeeding, and through under- and over-dilution, under- and over-feeding, infection resulting from unhygienic preparation and/or microbial product contamination, and other forms of industrial contamination (e.g. China’s 2008 melamine poisoning crisis) [8, 24–26]. Indeed for decades, ‘bottle-baby syndrome’ – a cycle of diarrhoea, dehydration and malnutrition resulting from artificial feeding in less than ideal conditions – has been reported in many countries [6, 27]. In 1939, in her now famous speech *Milk and Murder*, the paediatrician Cicely Williams reported on deaths resulting from ‘misguided propaganda on infant feeding’ [27]. In the 1960s, the aggressive marketing and promotion of BMS contributed to precipitous declines in breastfeeding in many countries, widespread ‘commerciogenic’ malnutrition of the child, and potentially millions of deaths [27, 28]. This triggered worldwide public scrutiny in the early-1970s, and later the birth of a transnational advocacy network – today the International Baby Food Action Network (IBFAN) – and what was to become the largest ever consumer boycott in history, against Nestlé the global market leader. Facing a public relations crisis at the time, in 1975 eight companies under Nestlé’s leadership established a lobby group – the International Council of Infant Food Industries (ICIFI) – and so began the industry’s organized efforts to counter its public health opponents [27–29].

The late 1970s was also a time of accelerating globalization, and calls for new forms of international regulation, to hold increasingly powerful transnational corporations accountable [30, 31]. The International Code of Marketing of Breast-milk Substitutes (The Code) [32, 33], was the first such code adopted under the auspices of the UN system, with WHO and UNICEF staff leading the stakeholder consultation and drafting process [27, 28]. Throughout this process, ICIFI and governments supporting the industry, lobbied to weaken The Code’s legal status, scope of provisions and wording [27, 28]. Despite this opposition, the World Health Assembly (WHA), as the world’s highest health policy-making body, adopted The Code in May 1981, with 118 member states voting in favour, three abstaining, and the US the single vote against. As the WHA resolution passed spontaneous applause erupted, and from the public gallery overlooking the plenary room a baby began to cry – a reminder to the assembled delegates ‘of what was at stake’ [27]. Importantly, implementation and monitoring of The Code is supported by the United Nations Convention on the Rights of the Child, and its monitoring body the Committee on the Rights of the Child [34]. The Code is a living document, strengthened biannually through WHA resolutions, in response to evolving industry practices and WHO technical guidance [27].

The Code’s adoption was a laudable public health success. However, its worldwide implementation has since faced sustained industry resistance, and 40 years later, there is still a long way to go. According to the latest monitoring report, 136 of 194 reporting countries (70%) have adopted at least some provisions of The Code into national law, but just 35 (18%) have adopted all provisions, and 58 (30%) have no legal measures whatsoever [35]. Furthermore, in-spite of The Code, milk formula markets have massively expanded since 1981. In earlier studies, we describe this expansion as representing a global infant and young child feeding transition to diets higher in commercial milk formulas [8, 24]. This transition reflects transformations in the systems that structure feeding practices at the population level – what we call *first-foods systems* [36, 37]. Such transitions and first food systems transformations are not new phenomena. Precipitous declines in breastfeeding and the normalisation of formula-feeding in many countries throughout the mid-twentieth century, was linked with among other things, income growth, urbanization, the shift in women’s work outside of the home, processes of medicalization, and intensified commercial marketing [37].

The transition we are now observing is, however, different for several crucial reasons. First, the scale-of-change is unprecedented. Growth in formula-feeding is occurring predominantly in industrialising middle-income countries, home to the world’s largest child populations. Between 2005 and 2019 alone, the world sales volume more than doubled from 1 to 2.2 million tonnes per annum, a rate that far exceeds changes in the global birth rate [38]. Second, it is occurring in the context of continuing economic globalization, including rapid growth in the size, transnational reach and consolidation of the baby food industry, with the large majority of sales accruing to just a small number of ‘Big Formula’ corporations [37, 39]. These corporations are reportedly using intensive and sophisticated marketing techniques to reach mothers, and to grow their markets on a global scale [39–41]. Despite these developments, surprisingly little attention has been paid to understanding the globalization of this industry, nor the market and political strategies Big Formula uses to expand, sustain and protect its markets, with some exceptions [42–45]. The role of the wider ‘baby food industry’, including dairy and other ingredients suppliers, advertising and public relations agencies and so on, throughout the supply chain, is also not well understood.

In this paper we address key questions. Who is Big Formula and the transnational baby food industry? How has this industry evolved, and how is it now organized across markets and globally? What strategies has the industry used to shape first-foods systems, and in doing so, drive milk formula consumption on a global scale? How can we understand the market and political practices of the industry in terms of power, and in doing so, inform new modalities of public health action?

## Materials and methods

Although the literature on Big Formula’s marketing practices is extensive, there are limited studies on the wider market and political practices of the industry. We therefore adopted a synthesis review method that allowed us to draw from extant literature, but also to integrate new qualitative and quantitative data to address gaps in knowledge. This involved several steps: i) development of a theoretical framework to guide the study; ii) search for relevant academic and grey literature; iii) data collection and descriptive statistics; and, iv) development of themes and synthesis of final results.

### Theoretical framework

We have defined and described the main components of first-foods systems in our earlier work [36, 37]. To understand the power of the baby food industry to shape first-foods systems, we integrated concepts from the CDOH and political economy of food systems literatures (Table 1) [4, 46–49].

**Table 1:**
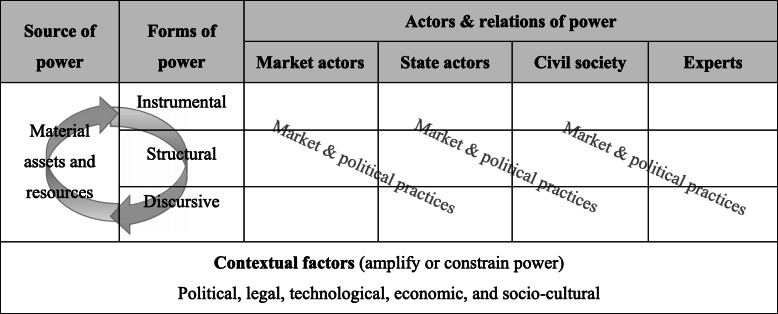
Theoretical framework used to understand corporate power and guide the study

First, we defined corporate actors, something often missing in CDOH scholarship. ‘Big Tobacco’ is often used as a collective term for the world’s largest tobacco manufacturers. Similarly, we used ‘Big Formula’ to refer to the corporations that manufacture and distribute BMS on an industrial scale, most but not all, being transnational corporations with a market presence in two or more country markets. We also viewed each corporation as anchored in their country of origin, and hence as identifying with nationally-derived cultures, operational structures and relationships with their home country governments [50, 51]. The ‘baby food industry’ comprises Big Formula at its core, but also the dairy industry and other input suppliers, retailers, advertising agencies, and various other commercial entities who profit from BMS [21, 23].

Executives and senior management run corporations, with a fiduciary duty to maximise profit, and through sustained profit, generate returns to shareholders (the owners) [50, 51]. To realise this interest, every effort is made within the legal constraints the corporation operates under, and sometimes beyond these constraints, to externalize as much of its costs of production as possible. The functioning of the market economy ensures these costs (or in economic terms ‘externalities’) are in the public domain, and so must be addressed by governments, or absorbed by social groups (e.g. higher morbidity, and health care costs) and/or the environment (e.g. water pollution or greenhouse gas emissions from dairy production). In pursuit of their interest, corporate actors seek to minimise conflict, neutralise or co-opt other societal actors, be they market (e.g. consumers, competing firms or suppliers), state (e.g. governments and inter-governmental organizations), civil society (e.g. non-governmental organizations, social movements and the media), and expert (e.g. scientists, academics and health professionals) actors [52].

Scholarship on the tobacco, alcohol and ultra-processed food industries often refers to a set of market and political practices (i.e. applied strategies and tactics) used to influence other actors within the system [2, 46, 53]. We organized these same practices under several overlapping and reinforcing concepts of power.

Arguably, the main source of corporate power is material, referring to the assets and resources acquired by corporations over time [47, 54]. With regards to Big Formula, we considered inter alia their sales revenues, profits, finance, productive assets (e.g. factories), human resources, trademarks and proprietary technologies among others. As corporations grow and globalize, these accumulating assets and resources can be readily converted into instrumental, structural and discursive forms of power. Instrumental power is the power to influence others directly [47, 48, 55]. For example, we anticipated that corporate executives may be members of elite social networks, with direct access to political leaders and government officials. Furthermore, that Big Formula uses its resources (and also ‘pool resources’ across the industry) to hire lobbyists, lawyers and public relations firms, make political donations, recruit former governmental officials, finance front groups and think tanks, form business coalitions, employ large sales forces to engage health professionals, and so on.

Structural power is the power to shape agendas and control the behavioural options available to others, without taking direct action [48, 49, 55]. For example, governments might make regulatory concessions to attract (or retain) the investments and employment opportunities Big Formula provides. In a strategy known as policy substitution, corporations might adopt voluntary private standards to delay or even replace regulation by the state; or support public-private partnerships (PPPs), that expand corporate influence in defining policy agendas and decision-making. As markets become more consolidated, Big Formula might exert greater power over suppliers to reduce costs (i.e. oligopsonistic power), control the product types and prices available to consumers (i.e. oligopolistic power), and thereby maximise its profit margins. Discursive power is the power to shape attention, influence (or supress) knowledge and evidence, and frame debates [48, 49, 55]. It is the power to socialise others, often unconsciously, into accepting certain problem interpretations and behaviours as normal, acceptable or socially desirable. To this end, we anticipated that Big Formula might finance public relations initiatives, attempt to shape scientific processes and wider knowledge environments, and engage in sophisticated forms of marketing.

We viewed these forms of power as interacting. For example, to counter regulatory threats, lobbyists may coordinate their discursive strategies across multiple decision-making spaces simultaneously; private standards can be both a form of structural power by substituting for regulation by the state, and discursive by portraying corporations as responsible social actors; marketing not only influences and drives consumer behaviour, but also socialises health professionals, policy-makers and others into adopting pro-industry beliefs. Wider contextual factors support or constrain corporate power, including the political, legal, technological, economic, and socio-cultural structures and systems in which they operate [56, 57]. For example, we anticipated that trade and investment liberalization has enabled Big Formula’s global expansion, including its cross-border supply chains, while the expanding scope and depth of trade agreements has constrained the ‘policy space’ of governments to regulate formula markets within their borders [19, 58]. Inadequate paid maternity leave entitlements in many countries, enables Big Formula’s power, by making formal maternal employment less compatible with breastfeeding. We viewed a major constraint on the power of Big Formula as the norm-promotion and accountability work of civil society groups (e.g. IBFAN), international organizations (e.g. WHO, UNICEF) and others [37, 59].

### Qualitative data collection

To source existing literature, we applied a semi-systematic branching search strategy, considered appropriate given the complexity of the topic, and the need to discover and draw from diverse literature sources.

First, we searched scholarly and web databases with comprehensive coverage of health, economic and social science sources, including PubMed, Scopus, Web of Science, EconLit, Eldis, Google Scholar and Google. We used relevant IYCF-related search terms (e.g. breast milk substitute*, formula*, breastfeed*), combined with actor-related (industr*, compan*, corporat*, commercial*, government*, state, civil society), and policy and practice-related (e.g. politic*, policy, marketing, advertising, promotion, public relations, lobby*, donation*) terms, with no date limits.

Second, to source grey literature, we searched the websites of WHO, UNICEF, FAO, UNSCN, Codex Alimentarius Commission (CAC), World Bank and World Trade Organization; and the civil society organizations IBFAN, Helen Keller International, FHI 360 / Alive & Thrive and Save the Children. We sourced industry reports from Euromonitor Passport, and from company and trade association websites.

As our understanding of the topic evolved, and reference lists were examined, further branching searches were conducted until we reached saturation (i.e. minimal new data was found with each additional search). This iterative process resulted in further discovery of media articles and internet sources.

Documents were included if published in English, relevant to the study aim, with described objectives, a clear method (if applicable), and conclusions substantiated by the findings.

### Quantitative data collection

Quantitative data was collected from diverse sources.

To describe the material assets and resources of companies, we sourced data from market databases, triangulated where possible with data from company websites and annual reports. Data on milk formula sales volumes (kilograms) and values (US$ at fixed exchange rates and current prices) for the years 2005–18, and data on market share (% market sales attributed to global company) for the years 2010–18, were sourced from the Euromonitor Passport database, for the world’s largest 78 country markets [60]. We have described this data extensively elsewhere [19, 24]. Data on total company sales, profits and assets, global rankings, and employee numbers were sourced from Compustat Industrial [61], Fortune 500 [62], and Forbes Global 2000 [63].

To understand the industry’s evolving global production and distribution networks, we used milk formula sales data from Euromonitor Passport, and sourced trade flow (imports and exports in US$) data from UN Comtrade [64], using HS Code 190110 for the years 2005–17. We then generated milk formula production estimates by adding the net-export value to the total sales value for each country/year (given total sales = production - exports + imports). To understand the industry’s evolving sourcing networks, data on dry milk powder production values (tonnes) were sourced from FAOSTAT [65], using codes 897 and 898, and trade flows (imports and exports in US$) from UN Comtrade using HS Codes 040221, 040229, and 040210.

To understand Big Formula’s global network of trade associations (i.e. lobby groups), we sourced initial ‘seed’ data from trade association membership disclosures listed on company websites, and then sourced additional membership data from those trade association websites, further snowballing until no new data was generated. We recorded ‘membership’ as reported on websites at the time of data collection, and hence this may data may not represent actual membership at the time of publication, nor can we validate the accuracy of content sourced from these websites.

To better understand the role of industry in shaping global regulatory standards for BMS at the CAC, we enumerated the number of member state delegates and observers from governments, international organizations, industry groups and public-interest non-governmental organizations attending the Codex Committee on Nutrition and Foods for Special Dietary Uses (CCNFSDU). We extracted data on the listed affiliations and/or email addresses found in CCNFSDU meeting agenda documents, available on the CAC website [66].

Early in our investigation it became apparent the US Government, representing the interests of the US-based corporations and dairy industry, has had a disproportionate influence in shaping relevant international standards, and constraining worldwide implementation of The Code. To further understand the lobbying power of the corporations in the US, we sourced data from the Centre for Responsive Politics [67]. This included total lobbying expenditures (US$) by market leading corporations, for the years 1998–2019, and US Government branches and agencies targeted by this lobbying; and lobbying expenditures (US$) declared as BMS- or trade-related, and the number of lobbyists employed, for the years 2007–18.

### Analysis and synthesis

All documents were uploaded to the qualitative analysis software NVivo (QSR International) and, guided by the theoretical framework, coded using constant comparative analysis by the lead author. This involved establishing, integrating and/or adding to the coded concepts over several iterations of coding the documents [68]. Given the large number of sources used, and the complexity of the topic under study, we did not use multiple-coders nor assess coder reliability. These techniques were used more to organize and retrieve the qualitative data, and to develop and refine emergent themes. We generated descriptive statistics using Excel (Microsoft) and R version 3.6.2 (Foundation for Statistical Computing). Network graphs were generated using Gephi version 0.9.2 (Association Gephi). Finally, we synthesised the qualitative and quantitative data into a final set of themes, which are presented in the results.

## Results

The following section presents the results, organized into key themes. We did not find significant recent literature on this topic. Therefore, in many places we draw upon and present new empirical findings.

### The rise of big formula and the material foundations of its power

In this section we describe the evolution of the baby food industry, and in doing so, describe the material assets and resources the corporations have accrued, as milk formula markets have expanded worldwide.

Today, Nestlé, Danone, Reckitt Benckiser Mead Johnson (RBMJ), Abbott Laboratories (Abbott), Friesland Campina (RFC) and Feihe are the global market leaders. Table 2 provides a breakdown of their assets and resources. Although pharmaceutical companies have historically dominated the industry, especially in the US and reflecting the unique nature of the product ‘on the dividing line between food and pharmaceuticals’ [21], Big Formula now spans the pharmaceutical, food manufacturing and consumer goods sectors. The top-five are extensively globalized. Nestlé, Danone, Abbott and RFC are present in > 100 country markets and RBMJ in 50, with affiliate or subsidiary firms in most. Nestlé has a near ubiquitous global presence. With the exception of Feihe, which operates in China alone, Big Formula are transnational corporations headquartered in Europe or the US. In 2016 all corporations, with the exception of Abbott, generated the majority of sales in emerging markets (Table 2).

**Table 2:** Material assets and resources of the world’s largest Big Formula corporations

Corporation	Nestlé (Gerber / Wyeth)	Danone (Nutricia)	Reckitt Benckiser (Mead Johnson)	Abbott Laboratories (Abbott)	Royal Friesland Campina	Feihe
*General*						
Headquarters	Switzerland	France	USA / UK	USA	Netherlands	China
Sector(s) of origin	Food manufacturing	Dairy / Food manufacturing	Consumer goods	Pharmaceuticals	Dairy / Food manufacturing	Food manufacturing
Year founded	1867	1919	1905^γ^	1888	1879^δ^	1962
Global 500 ranking (2018)	76	426	–	103	–	–
Total assets (US$ millions) 2018	139,244	52,096	44,399	67,173	10,403	1791
Total intangible assets (US$ millions) 2018^α^	51,155	28,828	35,705	42,196	2014	7
Total sales (US$ millions) 2018	93,242	29,070	14,855	30,578	13,940	1,570
Profits (EBITDA) (US$ millions) 2018	18,475	5276	4363	7562	889	432
Number of employees (foreign)	308,000 (298,000)	105,783 (98,378)	42,400 (38,746)	~ 103,000 (−--)	23,816 (~ 16,000)	–
*BMS-specific*						
World BMS market share % (retail sales value) 2018 (2010)	17.9 (13.8)	13.5 (12.1)	10.6 (12.9)	9.7 (11.5)	5.1 (2.7)	4.8 (1.4)
World BMS sales value ($US millions) 2018 (2010)	9373 (3567)	7093 (3126)	5579 (3340)	4097 (2985)	2796 (706)	2505 (354)
BMS sales as % of total sales 2018	10.0	25.5	33.7	13.4	20.8	–
Number of countries with products	190	120+	50	160+	100+	1
BMS brands sold worldwide	22	18	10	7	5	1
BMS brands with > 1% world market share^β^	NAN, Illuma, S-26, Nido	Aptamil, Nutrilon, SGM	Enfamil, Enfagrow	Similac, Pediasure, Eleva	Friso	Firmus
% sales from emerging markets 2016 (% from developed markets)	71.5 (28.5)	65.3 (34.7)	52.3 (47.7)	42.6 (57.4)	92.8 (7.2)	100
Markets with subsidiary / affiliate firms	114	77	28	89	36	–
# employees in BMS division	–	21,000	–	–	2348	–

Notes: Financial data sourced from Compustat, Fortune 500, Forbes, company annual reports and financial statements; data on compliance with The Code from Access to Nutrition Index; market share and sales data from Euromonitor Passport; currency conversions were made using 2018 average currency rate for the relevant financial year; α = intangible assets refer to non-physical assets, including brand recognition and intellectual property, such as trademarks, copyrights and patents; β = most brands include standard, follow-on, toddler and specialised milks under the same name; γ = founding date of Mead Johnson, acquired by Reckitt Benckiser in 2017; δ = the parent companies Friesland Foods and Campina merged in 2008, but were founded in 1879 and 1979 respectively

None sell BMS exclusively, comprising between 10 and 33% of total sales. However, the category has been a major, and if not the main, source of new revenue growth. For example, in 2018 the Nestlé Nutrition and Health Science division was the second largest contributor to total global sales, but the most important for generating new sales growth [69]. Big Formula also includes other transnationals such as Kraft Heinz (US) and Groupe Lactalis (France), and important regional players such as Hipp and Hero Group in Europe. National firms are leaders in several markets, for example, Vinamilk in Vietnam, Meiji and Morinaga in Japan, and Namyang in South Korea. China is home to several large home-grown corporations [38]. Big Formula are major employers, with the largest-five employing ~ 580,000 people between them worldwide. For Nestlé, Danone and RBMJ, only a small fraction (< 10%) are in their home countries. Approximately one fifth of Danone’s workforce, and one tenth of RFC’s, are employed in the division that manufactures BMS.

Until the mid-nineteenth Century babies were breastfed, or due to the mother’s death in childbirth, for other medical reasons or by choice, they were often wet-nursed by another woman. In some contexts, wet-nursing was an organized and regulated profession. In others, it was a service provided by family members, by slaves for their masters, or by poor women for the rich [6, 70, 71]. Artificial feeding of animal milks or other liquid foods also occurred, however it often resulted in malnutrition and high infant-mortality, exacerbated by poor sanitation and food hygiene [71, 72]. It was in this context the German chemist Justus von Liebig patented the first milk formula in 1865, informed by studies on the chemical composition of cows and human milk. By 1869, *Liebig’s food for infants* was being sold in Europe and the US, made from cow’s milk, malt and wheat flour, and potassium bicarbonate, and available first in liquid and then in powdered form, and purchased mostly by the wealthy. New techniques and materials for manufacturing bottles and teats, helped to promote the normalisation of artificial feeding, and supported early market expansion. By 1883, at least 27 patented brands of infant food had become available, and the age-old profession of wet-nursing quickly declined [70, 71].

Henri Nestlé, the founder of the company bearing his name, pioneered many of the industry’s early marketing techniques, including ‘direct mail’ of brochures to new mothers, and a ‘medical strategy’ of engaging doctors, conducting clinical trials, advertising in medical journals, and product endorsement by prominent scientists and health professionals [73]. Nestlé, and other companies like Britain’s Cow and Gate, were at the vanguard of the industry’s first-wave of globalization, expanding along European colonial pathways, and benefiting from their ‘first-mover advantage’ in many markets [74, 75]. By the 1920s, Nestlé was by far the market leader, with 80 factories operating worldwide, plus 300 sales offices, depots or agencies [76]. By this time, most of today’s commercial milk formula brands had become available [27, 71]. This included specialised milks for certain medical conditions affecting a small proportion of infants, the first using soy-based protein for those allergic to cow’s milk [77]. Milk formula markets steadily expanded throughout the mid-twentieth century, alongside more intensive marketing to health professionals, the medicalisation of pregnancy and birth (including the frequent separation of mother and infant in birthing clinics), and the widespread use of formula in hospitals. These developments coincided with a precipitous decline in breastfeeding in many countries, reaching historic lows in the 1960s-70s [71].

From then onwards, however, breastfeeding rates began to resurge in many of Big Formula’s markets. This along with declining birth rates following the post-World War II ‘baby boom’, resulted in stagnating sales, and in response, companies started to intensify their marketing practices in markets of the Global South [27, 52]. Marketing techniques used to promote and normalise formula-feeding, included mass-media advertising, large-scale distribution of free samples, and salespeople dressed as ‘mothercraft nurses’, to engage mothers directly in maternity wards and in their homes [27, 28]. These practices were soon associated with widespread ‘commerciogenic malnutrition’ and infant deaths [6, 28], which in-turn triggered worldwide civil public scrutiny, and events that would lead to the adoption of The Code in 1981. Despite these developments, market expansion continued apace. Between 1978 and 1983, total world sales nearly tripled, from ~US$1.5 billion to ~US$4 billion [78], mainly through exports to overseas markets, as Big Formula took advantage of subsidies for dry milk products in the US and Europe [29]. In 1984, facing new marketing regulations promulgated by The Code, the industry began marketing more intensively a wider range of product categories for older infants and young children. The availability of follow-up (6–12 months) and toddler (13–36 months) formulas markedly increased [27, 79], and in many markets today these categories now represent a near-equal, or even greater, market share than infant formula [80].

The Code was, in some respects, a product of its time. The 1970s–80s was an era of accelerating globalization, with rapid growth in the number and size of transnational corporations, and their economic power relative to nation states [30, 51]. With this came vocal calls from civil society, many governments, and various UN agencies, for the internationalisation of corporate regulation, with The Code being one among ~ 30 such codes and guidelines proposed across the UN system at the time [30, 31]. Remarkable changes in the global political and economic system have occurred since then. In the 1980s, the rise of neoliberal economic and social policies led to market liberalization, privatization and growing preferences for market-based approaches to governance [81, 82]. A ‘corporate food regime’ emerged, as transnational food corporations, mostly through foreign direct investment, began to globalise with renewed vigour, seeking growth opportunities in the rapidly industrialising countries of the Global South [48, 83, 84]. The establishment of the World Trade Organization (WTO) in 1995, and then an explosion in free trade agreements, accelerated this process, allowing such corporations to integrate their ‘global value chains’, while imposing new rules on how governments regulated their markets [85–87].

**Fig. 1:**
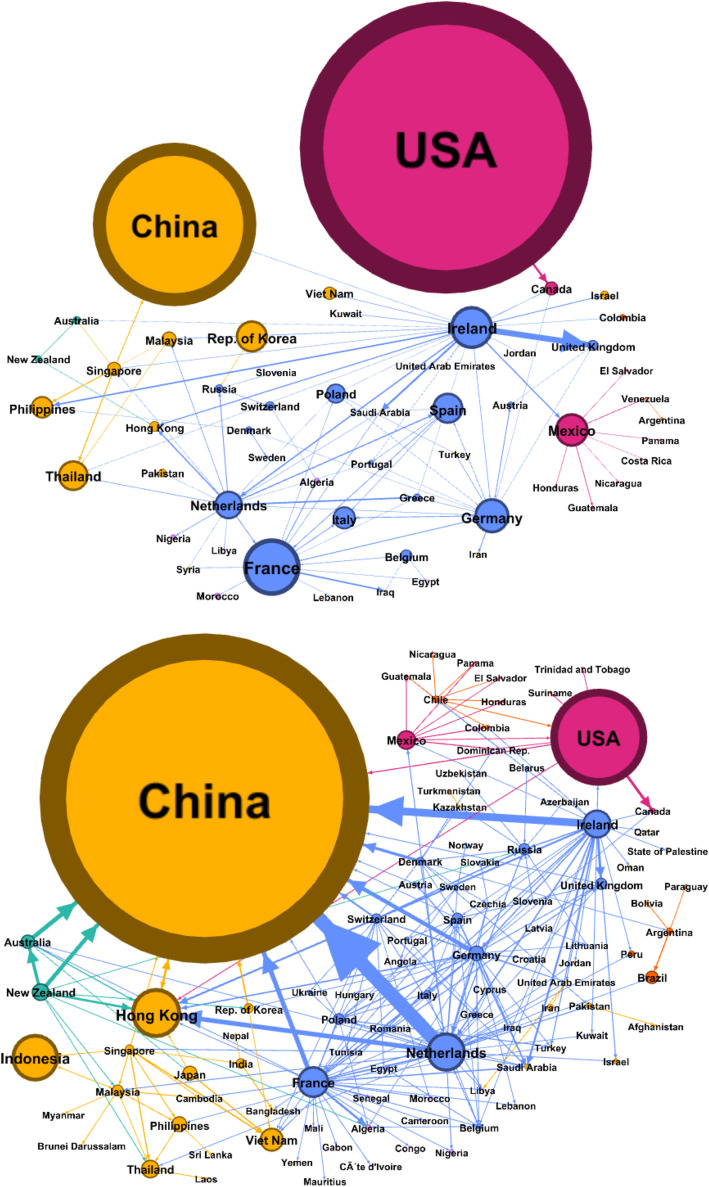
Changes in global milk formula production and trade flows (US$), showing 2005 (top) and 2017 (bottom); circles represent country production values and lines the value and direction of trade. Notes: To simplify the figure, only countries with trade flow values >US$5 million were represented; milk formula sales data were sourced from Euromonitor Passport; trade data were sourced from UN Comtrade

The above developments fostered ripe conditions for the most recent and remarkable phase of Big Formula’s global expansion. As shown in Fig. 1, this is reflected in the massive expansion in worldwide milk formula production and trade flows. In 2005, only Ireland and Singapore were exporting >US$5million of milk formula for retail sale to China; by 2017 at least 16 countries were – most notably Australia, New Zealand, France, and the Netherlands. In 2005, total world sales were US$22.9 billion; by 2019 this figure had more than doubled to US$55.6 billion [37, 38]. The world sales volume per child (0–36 months), more than doubled from 3.5 to 7.4 kg over the same period. This growth occurred mostly in the industrialising and highly-populated middle-income countries of East and South East Asia, and to a lesser extent Eastern Europe & Central Asia, Middle East & North Africa, and Latin America. During this period, China became the world’s largest and most competitive market. In 2005, the US was the world’s largest market and China represented just 14.1% of global sales. By 2019, it represented 32.5%, 2.3-fold larger than the US and Western European markets combined [38].

Today Big Formula is ‘hyper-globalized’ [87], with extensive global sourcing and production networks. In 2018, for example, Nestlé had 443 factories operating across 80 countries, of which 40 were listed as the division that manufactures BMS [69]. Abbott operated 27 production sites globally, of which 14 were listed under its Nutrition division [88]. Market expansion has also been enabled by massive growth in the industries providing milk formula manufacturing inputs. Between 1961 and 2014, for example, production of dry milk powder grew from 491,000 to 3,444,000 t, initially from output in European countries, and then from countries in Australasia and Latin American with industrial dairying systems [89, 90]. Figure S1 shows the significant expansion in dry milk powder production and trade flows between 2005 and 2014. Markets for dry milk powder are dominated by a handful of ‘Big Dairy’ corporations, including for example, Fonterra (New Zealand), Dairy Farmers of America (US), and Groupe Lactalis (France). China’s formula boom was enabled by expanded dairy production and exports from New Zealand in particular, which expanded rapidly following the Free Trade Agreement signed between the two countries in 2008. New Zealand was the first country to sign such an agreement, and by 2012, dairy exports comprised 30% of the country’s total exports to China [91, 92]. In recent decades vegetable oil production, and especially palm oil as a common milk formula ingredient, has also significantly expanded [19, 93].

**Fig. 2:**
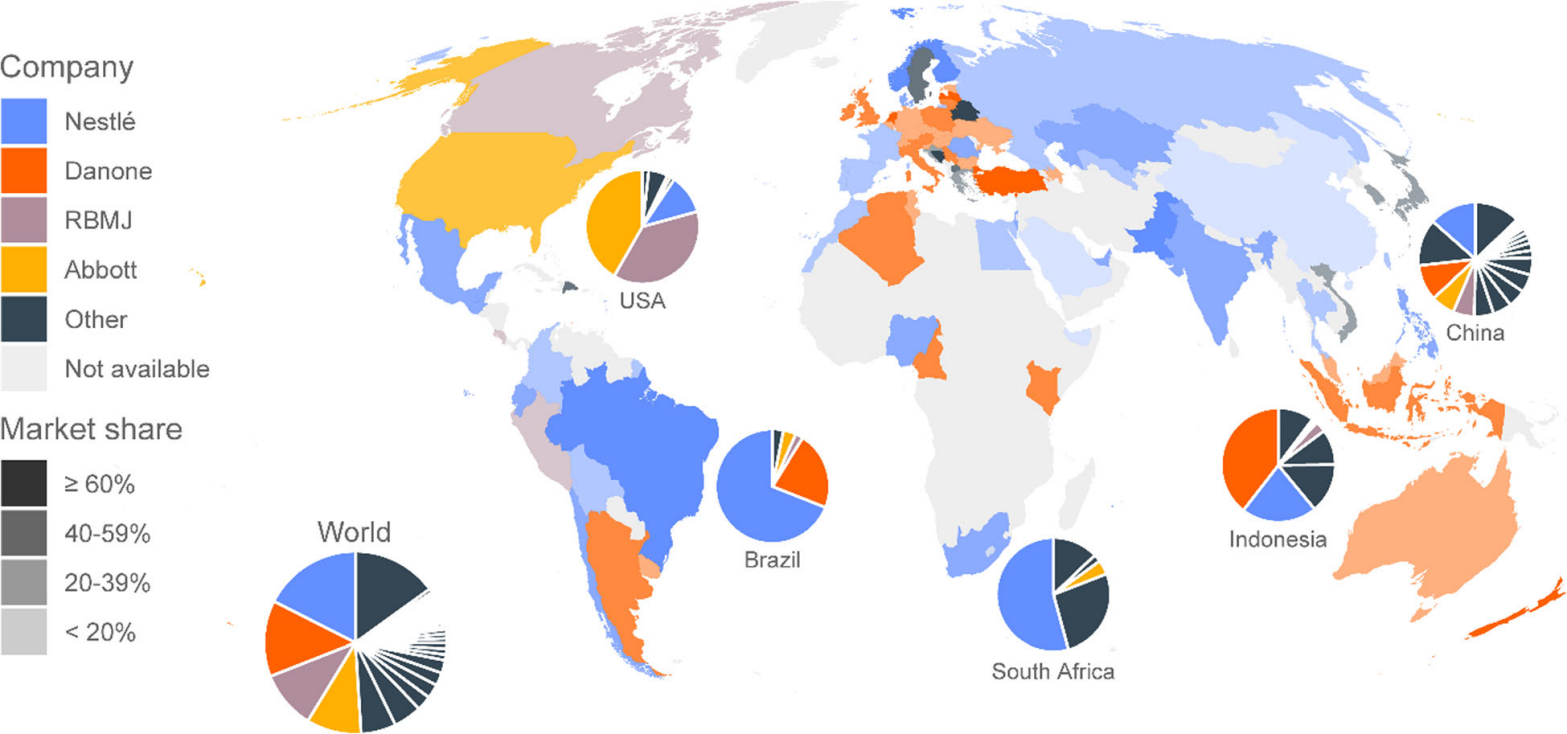
Market share (%) held by leading corporations in the world’s largest 80 milk formula markets. Notes: Data were sourced from Euromonitor Passport; interpret this figure by considering which corporation leads in each country market by colour, and the degree of shading indicating their % market share – for example, Nestlé leads in both China and India, but has a much higher market share in India; the pie charts show more detailed data of the % market share held by corporations in the world, and in key illustrative markets – for example the Brazilian market is highly concentrated, and dominated by Nestlé, followed by Danone, whereas in China Nestlé leads but the market comprises many more players

The rise of Big Formula’s material power is also reflected in high levels of market concentration nearly everywhere. Figure 2 shows the market shares of leading corporations in key country markets and worldwide. In 2018, 61.6% of world sales accrued to the six largest milk formula manufacturers listed in Table 2. The three largest – Nestlé, Danone and RBMJ – had a combined world market share of 42%. In 2010, the largest four had near equivalent sales, but since then Nestlé and Danone have further consolidated their market positions. These gains have occurred through organic sales growth, but also through sustained merger and acquisition activity, with the industry currently undergoing ‘terminal’ consolidation [21]. Recent acquisitions have included Danone’s of Numico/Nutricia in 2007 for US$18 billion, Nestlé’s of Pfizer’s infant nutrition division in 2012 for US$11.9 billion, and Reckitt Benckiser’s of Mead Johnson Nutrition in 2017 for US$16.6 billion. The acquired firms were already products of various mergers and acquisitions, going back many decades [21]. Regional and national markets are even more consolidated. As shown in Fig. 2, many are oligopolistic, for example in the US, Brazil, South Africa and Indonesia, where only a few corporations dominate. The exception is China, where Nestlé leads, but a diverse mix of transnational and domestic players compete [21]. Consolidation can enhance Big Formula’s market power over suppliers, allowing the sourcing of manufacturing inputs at lower-cost. By capturing markets, companies can also exert greater control over prices, and what products are available to consumers.

Several transformations in Big Formula’s distribution networks, also explain the phenomenal global expansion of milk formula markets. First, the liberalization of trade in retail services has enabled the ‘supermarketization’ of developing countries since the late 1990s, which alongside pharmacies, is a key channel for reaching urban consumers with rising incomes [19, 24]. Second, the medicalization of pregnancy, birthing and infant care in many countries, has created new opportunities for the industry to market products through health-care providers (see health professional co-optation), just as new practices that undermine breastfeeding, such as birth by caesarean section, have increased markedly in many countries [37, 94]. Finally, growth in ‘grey-market’ trade by third parties has also contributed significantly to rapid market expansion [24, 37]. Sophisticated Daigou (grey channel) operations in Australia, New Zealand, the UK and Germany have involved shoppers purchasing well known branded products for export in suitcases or small shipments to China, to the extent that in 2014, Daigou sales were equivalent to half of what foreign companies were selling in the formal Chinese market [42].

### The baby food industry’s global influence network

**Fig. 3:**
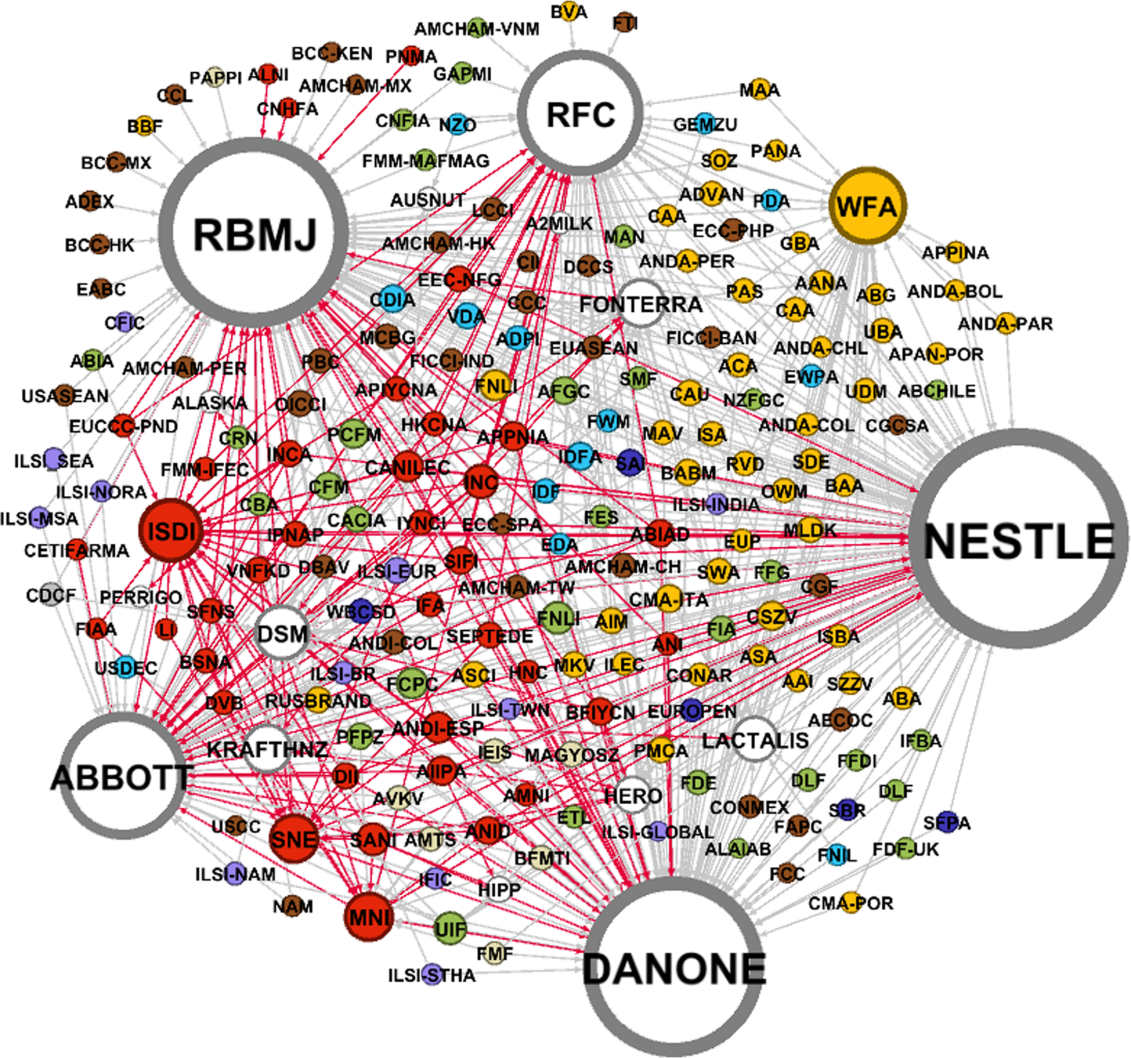
The baby food industry’s global influence network of trade associations and other corporate-funded influence organizations, with lines representing membership. Notes: See Table S1 for the full list of organization names and abbreviations; initial ‘seed’ data were sourced from membership disclosures listed on company websites and additional membership data then sourced from organization websites, further snowballing until no new data were generated. We recorded ‘membership’ as reported on websites at the time of data collection, and hence this data may not represent actual membership at the time of publication. As an example, RFC listed membership in 12 infant nutrition associations, 27 dairy associations, 21 food and beverage associations, five advertising associations, 19 business associations, 11 ‘other’ associations, and 14 collaborations and partnerships [95]. The size of the circles is proportionate to the number of ‘ties’ the organization has with others in the network; the white circles represent corporations in the baby food industry; the red circles and lines show Big Formula’s network of infant nutrition associations; yellow circles represent branding and advertising associations; green circles represent food, beverage and grocery manufacturers associations; brown circles represent general industry trade associations, for example chambers of commerce; light blue circles represent dairy industry trade associations; purple circles represent consumer information and industry-funded scientific organizations. This graph was generated using Gephi version 0.9.2 (Association Gephi)

To protect their worldwide interests, and to foster favourable regulatory and knowledge environments for expansion across their diverse markets, Big Formula and the wider industry employs an extensive global network of trade associations and other corporate-funded influence organizations. Figure 3 shows this network and Table S1 the full list of names and abbreviations. The lines represent membership in these organizations, which span many regulatory issues and corporate functions, at international, regional and national levels; the size of the circles represents the number of organizations each corporation associates with. The respective corporations are typically members of organizations in countries where they have a major market presence. Hence Nestlé, as the most transnationalised (Table 2), is a member of the most organizations in the network followed by Danone, RBMJ, Abbott and RFC. Major ingredients suppliers, such as DSM and Fonterra, also feature prominently.

Core to the network are ‘infant nutrition’ trade associations (red), which focus on baby food issues specifically. The first such organizations were established in the 1970s in response to emerging public relations and regulatory threats, enabling Big Formula to execute their public relations strategies and lobby at ‘arms-length distance’, while minimising negative publicity. The International Council of Infant Food Industries (ICIFI) was established by eight companies in 1975, following highly publicised Nestlé litigation against student activists in Switzerland [27, 28]. As civil society groups began to strongly agitate for adoption of The Code, ICIFI enabled Nestlé to make ‘third party rebuttals of the activists’ case’ [96]. However, because of this, ICIFI’s public reputation soon diminished, and it was replaced in 1984 by the International Association of Infant Food Manufacturers (IFM) [97, 98].

The IFM itself was disbanded in 2016 and today the International Special Dietary Industries (ISDI) is the industry’s peak international lobby group, with 20 member associations across six continents [99]. Two of these associations are regional – Specialised Nutrition Europe and The Asia Pacific Infant and Young Child Nutrition Association. Some have authoritative names, akin to professional and non-commercial organizations – for example, the Infant and Pediatric Nutrition Association of the Philippines, the Infant and Young Child Nutrition Council (India), and the Infant Nutrition Council of Australia & New Zealand. Some provide extensive infant and young child feeding advice on their websites. Others have developed clinical standards and guidelines for infant care. For example, Abbott provided seed funding to establish the European Foundation for the Care of Newborn Infants (EFCNI), ‘to represent the interests of preterm and newborn infants and their families’ [100]. Hence, these ‘front groups’ appear to be public-interest civil society organizations, but in fact represent corporate interests [46].

Figure 3 shows the influence network also includes trade associations and lobby groups concerned with many other corporate issues and regulatory affairs. In yellow, for example, is a network of advertising and branding associations, many of which are member organizations of the World Federation of Advertisers (WFA). The activities of these organizations are diverse, but mainly focus on protecting the intellectual property rights of corporate brands, promoting voluntary advertising codes, and lobbying against governments adopting mandatory marketing regulations. Other types also feature prominently in the network including general business associations like the US and European Chambers of Commerce, who seek to foster trade access and protect free enterprise (brown), food, beverage and grocery manufacturers associations (green), dairy groups like the International Dairy Federation (blue), and corporate-funded scientific institutes and communications platforms, for example the International Life Sciences Institute (ILSI) and the International Food Information Council (IFIC) (purple).

### Contesting standards in multi-lateral policy-making arenas

The lobbying activities of many organizations in the above network, are coordinated across multiple policy fora and decision-making spaces simultaneously. At the international level, this includes three key organizations that develop policy and govern the regulation of foods for infants and young children, and hence influence industry sales worldwide: the World Health Organization (WHO), Codex Alimentarius Commission (CAC), and World Trade Organization (WTO).

Technical standards and norms established by the WHO, are crucial in guiding global infant and young child feeding (IYCF) policy actions. Table 3 provides notable examples of how Big Formula, and member states representing the industry, have lobbied to undermine the scope and strength of The Code, since it was first proposed in 1979. Article 19 of the WHO Constitution grants the World Health Assembly, as the world’s highest health policy-making body, the power to adopt (listed from strongest to weakest) conventions, regulations and recommendations. As an example, the Framework Convention on Tobacco Control (FCTC) was adopted by the World Health Assembly in 2003, as a legally binding treaty. The adoption of The Code as a recommendation, rather than a more binding regulation, was attributed largely to the opposition of the US and other large-dairy producing member states at the time [27]. Since then, lobbying efforts have focused mainly on limiting the ‘regulatory scope’ of WHO technical guidance and subsequent WHA resolutions. This includes opposing the extension of the recommended duration of exclusive breastfeeding, and technical guidance concerning cross-promotion, and the designation of products for ages 6–36 months (i.e. follow-up and toddler milks) as BMS [44].

**Table 3:** Actions by or on behalf of the baby food industry in relation to key multi-lateral organizations that govern the regulation of foods for infants and young children

Targeted organization	Objective (inferred)	Description
World Health Organization	Weakening the initial scope and strength of The Code	In 1980, ICIFI hired Stanislaus Flache, a former assistant director-general of WHO, in order to gain insider knowledge and lobby WHO officials during the consultation and drafting process. Flache was quoted as stating ‘We oppose the universal code and some believe it is a sign that the UN system is moving to control multinationals’ [28]. ICIFI worked to dilute language during the drafting process [29]. A letter was sent to members of the WHO Executive Board, who were meeting in January 1981 to approve a draft document, stating the ‘World Industry has found this present draft code unacceptable…highly restrictive...irrelevant and unworkable’ and that ‘various provisions…could have a negative effect on child health’ [28]. In April, ICIFI circulated another letter stating the draft was ‘too detailed, counterproductive and, in parts, incompatible with the constitutional requirements of a number of countries’ [44]. US officials were engaged to lobby other member state delegates and WHO staff on the industry’s behalf. Major dairy-producing countries rejected initial drafts, including the US, Denmark, France, Netherlands, New Zealand and Switzerland. The adoption of The Code as a recommendation, and not as a stronger regulation, was done to appease US opposition in particular, which financed ~ 25% of the WHO’s regular budget at the time. The US, under the newly elected Reagan administration, was the only member state to vote against The Code in May 1981 [28, 29, 44].
	Delaying the extension of the recommended duration of exclusive breastfeeding	In 2000, the IFM (having since replaced ICIFI) unsuccessfully attempted to lobby WHO staff, to delay the adoption of new technical guidance and a WHA resolution planned for May that year, that would extend the recommended duration of exclusive breastfeeding from ‘4–6 months’ to ‘about 6 months’, and hence conceivably impact sales [44, 101]. This lobbying was coordinated across WHO’s six regional committee meetings that year, and the Executive Board meeting and WHA the following year [101]
	Opposing guidance on ending inappropriate promotion of foods for infants & young children	In 2016, IFM and ISDI issued a statement to the Executive Board to ‘manufacture doubt’ about new technical guidance clarifying The Code covered products marketed for ages 6–36 months, including follow-up and toddler milks, categories they considered outside of scope. Nestlé claimed because the final WHA Resolution 69.9 referred to the guidance as ‘welcomed with appreciation’ rather than ‘adopted or approved’, governments were not obligated to implement it [44, 102]. The resolution also called for an end to all forms of inappropriate promotion, as set out in the guidance, including cross-promotion. The International Dairy Foods Association (IDFA) endorsed the IFM and ISDI position, and engaged US officials to oppose the guidance, stating it was ‘alarmed by the non-transparent, flawed process by which the WHO has developed this guidance’ and called upon officials to ‘work aggressively toward improving the WHO’s processes and procedures to ensure the organization builds and maintains greater trust’ [44].
		In 2018, Trump Administration officials, aggressively opposed a new WHA resolution that included, among other provisions, the contested 2016 technical guidance. US Government delegates worked to water-down wording, questioned the supporting evidence, and threatened to remove military support and enact trade measures against Ecuador, the proponent of the resolution. This had a ‘chilling’ effect on others, with at least 10 member states declining to support the resolution, although it was eventually adopted by the WHA, through the leadership of Russia [44, 103].
	Challenging the WHO initiative on conflicts of interest in nutrition	In 2018, ISDI, the International Dairy Federation (IDF) and Global Dairy Platform, among others, provided submissions to a WHO consultation on a new tool for ‘Safeguarding against possible conflicts of interest in nutrition programmes’. The ISDI submission argued that managing such conflicts was best left to country governments. The IDF submission called into question the consultation process itself, requesting a postponement and wider consultation [104].
Codex Alimentarius Commission	Contesting revisions to Codex standards on infant, specialised and follow-up formulas	Contestations of the Codex Standard for Infant Formula and Formulas for Special Medical Purposes Intended for Infants, have included how The Code is referenced in the Standard, whether in the main text or as a lesser footnote; allowable ingredients, and minimum and maximum nutrient ranges; and the allowable nitrogen conversion factor for determining infant formula protein levels, with the IDF, supported by some dairy-producing member states, advocating for a higher value than the one proposed by leading expert groups [105, 106].
		Contestations of the Codex Standard for Follow-Up Formula have included the definition of products for ages 12–36 months, with pro-industry stakeholders arguing these are not BMS; advocating the use of the term ‘formula’ for products for young children, hence implying nutritional adequacy; whether to reference The Code and resolutions (like WHA 69.9) in the Preamble, with pro-industry stakeholders arguing sources ‘external to Codex’ should not be referenced; that neither additives with sweet taste, types of sweeteners, or sugar content should be restricted in the Standard; and that ‘cross-promotion’ is not clearly defined and should be excluded [105].
World Trade Organization	Countering country-level implementation of The Code, and fostering regulatory chill	Between 1995 and 2019, there were 110 interventions α made in the WTO concerning existing or proposed BMS marketing, labelling or safety testing regulations of a member state. The majority of these interventions occurred in the TBT Committee, mainly concerning whether regulations were more restrictive than international standards (including Codex standards), and considered scientifically justified. Interventions also occurred during periodic trade policy reviews, where member state policies were assessed for compliance with WTO agreements. An even greater number of interventions occurred during the screening of new members for accession to the WTO, with the large majority of countries undergoing the accession process experiencing either exploratory questions and/or issue-specific negotiations (Initial Negotiation Rights) [Russ K, Baker P, Byrd M, Kang M, Siregar RN, Zahid H, McCoy D: Understanding the global trade and public health regime complex: a case study on breastfeeding and commercial breastmilk substitutes. Forthcoming].

Notes: α = here the term ‘intervention’ does not refer to trade arbitration; rather, it is defined as questions or comments relating to BMS regulations or proposed regulations in one member state, registered to a WTO committee or council, or raised during a trade policy review, by another member state [Russ K, Baker P, Byrd M, Kang M, Siregar RN, Zahid H, McCoy D: Understanding the global trade and public health regime complex: a case study on breastfeeding and commercial breastmilk substitutes. Forthcoming]

Big Formula and the baby food industry have also influenced standard-setting processes at CAC, the UN food standard-setting body jointly administered by WHO and FAO, with a dual mandate to protect public health and safety, and to facilitate international food standards harmonisation and trade [30, 105, 107]. Codex standards – including specific ‘commodity standards’ for infant and specialised formulas, and follow-up formula, and ‘general standards’ on labelling, additives and others that apply to all commodities within scope – are developed by committees comprising voting member states, with technical input from observers, including industry trade associations and civil society organizations [105, 107]. Table 3 details several examples of how dairy-producing member states, and industry trade associations – mainly ISDI and the International Dairy Federation – have contested these standards.

This lobbying works in the interests of Big Formula and the wider industry for two key reasons. First, CAC standards function as a minimum benchmark, or regulatory ‘floor’, for the development of national regulatory measures on product composition, safety and labelling, and therefore influence regulatory standards worldwide [30, 107]. Second, the CAC is explicitly referenced in the WTO’s Sanitary and Phytosanitary Measures Agreement (SPS), and meets the criteria for a standard-setting body in the Technical Barriers to Trade Agreement (TBT). Subsequently, countries implementing regulatory measures more stringent than Codex standards may be required to provide scientific justification in the WTO and other trade policy fora. Because of this, CAC standard-setting processes have become highly politicised, with strong industry participation and influence [30, 107].

**Fig. 4:**
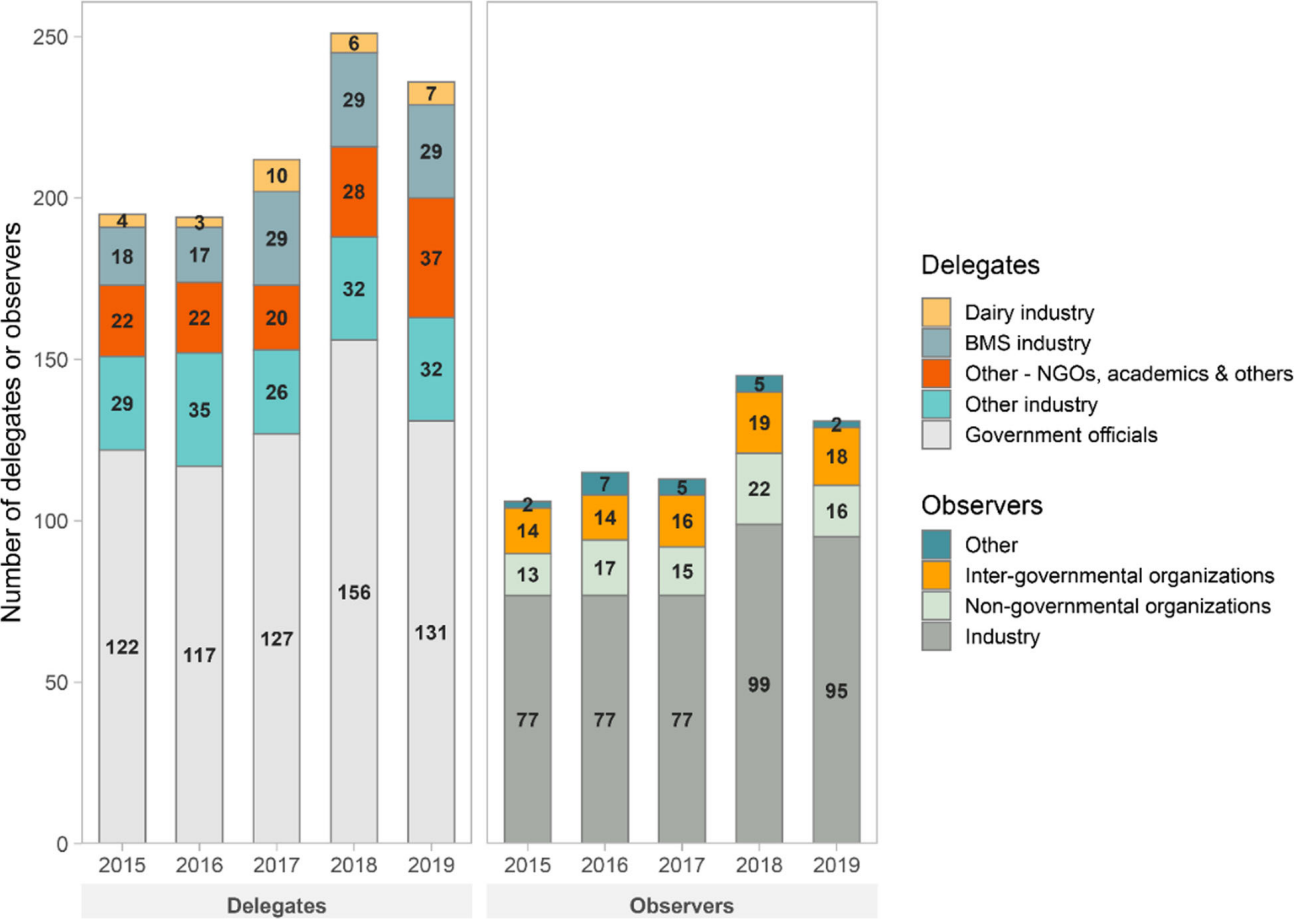
Affiliations of member state delegates and observers attending the Codex Committee on Nutrition and Foods for Special Dietary Uses, between 2015 and 2019. Notes: Data sourced from affiliations and/or email addresses listed for participants in CCNFSDU meeting agenda documents, available on the CAC website; note that email addresses for many participants were often obscure, hence the numbers in Fig. 4 are likely underestimates

In Fig. 4, we present new data on the affiliations of participants in the Codex Committee on Nutrition and Foods for Special Dietary Uses (CCNFSDU), mandated to develop the standards for infant and specialised formulas, and follow-up formula respectively. Between 2005 and 2019, industry not only comprised 70% of non-state observers participating in the CCNFSDU meetings, far out-numbering observers from civil society and inter-governmental organizations, but also 28% of member state delegations. In several instances industry representatives were the member state delegation in its entirety.

The influence of the baby food industry is also evident in the WTO, the inter-governmental organization for developing, maintaining and enforcing a global system of trade rules and agreements. As WHO and UNICEF recently reported, formal trade arbitration concerning national implementation of The Code (i.e. through a WTO dispute panel and settlement process) has yet to eventuate [108]. However, we have shown how member states with large dairy-producing industries – especially the US, EU, Australia and New Zealand – frequently use WTO processes to challenge BMS-related regulations adopted by other member states [Russ K, Baker P, Byrd M, Kang M, Siregar RN, Zahid H, McCoy D: Understanding the global trade and public health regime complex: a case study on breastfeeding and commercial breastmilk substitutes. Forthcoming]. Between 1995 and 2019, 110 interventions occurred against WTO member states relating to actual or proposed BMS marketing, labelling or safety testing regulations (Table 3). Here the term ‘intervention’ refers to ‘questions or comments relating to restrictions or proposed restrictions in one member country, registered to a WTO committee or council, by a delegation from another country, or during a trade policy review’. In some instances, interventions occurred across several years, resulting in significant changes to the planned implementation of The Code by a member state. For example, between 2015 and 2018, when Thailand started revising its ‘Milk Code’, including extending the scope of products from the ages 0–12 to –36 months, it faced repeated interventions via the WTO Trade Policy Review process, and then in the TBT Committee. The National Assembly passed the final legislation, but without the proposed restrictions on the marketing of products for ages 12–36 months [Russ K, Baker P, Byrd M, Kang M, Siregar RN, Zahid H, McCoy D: Understanding the global trade and public health regime complex: a case study on breastfeeding and commercial breastmilk substitutes. Forthcoming].

### Contesting standards in bi-lateral and national policy-making arenas

The baby food industry spend large sums on lobbying and political financing to influence government positions in the above international arenas, and to achieve favourable regulatory environments within countries. Several examples of this lobbying in national arenas, and also bilateral actions taken by governments against other governments on behalf of the industry, are listed in Table 4. Although Nestlé, Danone, RBMJ and Abbott all have corporate policies on lobbying, framed as ‘interactions with public authorities’, ‘advocacy’ or ‘political participation’ in Table 5 respectively, much of their lobbying is conducted by the aforementioned trade associations.

**Table 4:** Political actions by or on behalf of baby food industry in bilateral and national policy-making arenas

Country	Objective (inferred)	Description
Canada	Lobbying to influence a free trade agreement to resolve a non-tariff barrier to trade issue	Canada is the leading market for US processed dairy exports. In 2017, facing a surplus supply of skim milk, Canada implemented a new ‘Class 7’ milk price, making domestic products cheaper, and thereby increasing Canadian exports of skim milk powder, while making dairy product imports (including infant formula) from the US less competitive [109]. Between 2016 and 2019, this provoked US$6,184,614 in lobbying the US Government, by infant formula producers and dairy industry associations [67]. The US raised concerns with Canada bilaterally and in the WTO Committee on Agriculture. Eventually, it was agreed to eliminate the Class 7 price under the new US-Mexico-Canada Agreement (USMCA), signed in 2018. In addition, Canada was required to monitor its exports of skim milk powder and infant formula, impose a surcharge on exports exceeding thresholds specified in the USMCA, and expand its duty-free tariff rate quotas on US dairy imports [109]. [Russ K, Baker P, Byrd M, Kang M, Siregar RN, Zahid H, McCoy D: Understanding the global trade and public health regime complex: a case study on breastfeeding and commercial breastmilk substitutes. Forthcoming]
China	Delaying the introduction of new food safety regulations	China strengthened food standards through a new Food Safety Law in October 2015. This included stricter product safety regulations, harsher punishments for violators, strengthened accountability mechanisms including protections for whistle-blowers, product certification requirements, and new provisions for infant formula products [110, 111]. Between 2013 and 2016, the National Milk Producers Federation, Mead Johnson, Abbott, and Infant Nutrition Council of America spent US$1,255,577 on lobbying the US Government on this issue [67]. The US Government submitted comprehensive written comments on the draft measure, and also urged China to notify the draft measure to the WTO TBT Committee, and the WTO SPS Committee. Implementation of the new product certification requirement was delayed by 2 years [112]. [Russ K, Baker P, Byrd M, Kang M, Siregar RN, Zahid H, McCoy D: Understanding the global trade and public health regime complex: a case study on breastfeeding and commercial breastmilk substitutes. Forthcoming]
Guatemala	Challenging new labelling provisions	In 1983, Guatemala was among the first countries to implement The Code into national law. The Guatemalan Law on the Marketing of Breastmilk Substitutes Decree 66–83, and Government Agreement NO 841–87, mandated that all products must state breastmilk is the best food for children under 2 years of age, and prohibited the idealisation of formula through the use of pictures of infants. In 1992, the Gerber Company, which used a picture of the ‘Gerber Baby’ face trademark on its products, refused to comply with a request by the Food and Drug Registration and Control Division to comply with the law. Gerber requested a court injunction, claiming its products were out of scope, and that this violated intellectual property rights obligations under international trade law. Gerber engaged the US State Department to apply pressure on the Guatemalan Government to amend the labelling provisions under the law, threatening to remove Guatemala’s ‘Most Favoured Nation’ status under the US Generalized System of Preferences. In 1995, Guatemala’s Supreme Court of Justice ruled in favour of Gerber, arguing the law applied to locally produced products only, and not imported ones [27, 113].
Hong Kong	Preventing the expanded scope of marketing regulations	In 2012, the Hong Kong Infant and Young Child Nutrition Association, a trade association representing Abbott, Danone, RFC, Mead Johnson, and Nestlé, opposed a draft regulation that would ban the promotion of foods for children aged 0–36 months. A document was presented to legislators stating the legislation should follow The Code, and apply to products for 0–6 months only. It stated ‘There is no scientific evidence to show promotion of food for children 6 months or above has affected the breastfeeding rates and its duration…Any biased over-regulation in infant formula marketing will be contrary to Hong Kong’s open free market economy and…the fundamental right of consumers to information and choices.’ [114]. An extensive legal analysis was published, concluding the draft regulation violated the WTO’s TBT, SPS and TRIPS Agreements [115]. The US Trade Representative, in its 2017 report on foreign barriers to trade, stated ‘If the draft Code is implemented as originally drafted, U.S. stakeholders maintain that, together with related legislative proposals, it will have significant negative impacts on sales of food products for infants and young children, and is more restrictive than relevant international standards’. Furthermore, ‘The United States is continuing to engage with the Hong Kong government on this draft measure’ [116].
India	Minimising costs associated with mandatory product safety certification	In 2003, the Government of India strengthened its Infant Milk Substitutes, Feeding Bottles, and Infant Foods (IMS) Act, so that it bans the marketing of food for children up to 24 months of age, as well as marketing by BMS producers to medical professionals and organizations, enforceable with criminal penalties [117]. In 2009, the Government further revised its certification compliance list, which includes infant formula. Products on the list must be certified for safety by the Bureau of Indian Standards [118]. Between 2012 and 2014, Mead Johnson, Abbott, and National Milk Producers Federation spent US$2,435,240 lobbying the US Government on this ‘Indian Bureau of Standards regulatory issue’ [67]. [Russ K, Baker P, Byrd M, Kang M, Siregar RN, Zahid H, McCoy D: Understanding the global trade and public health regime complex: a case study on breastfeeding and commercial breastmilk substitutes. Forthcoming]
Indonesia	Requesting notification of new regulations to the WTO	In 2016, Indonesia’s food and drug regulatory agency, Badan Pengawas Obat dan Makanan (the National Agency of Drug and Food Control), issued a draft of the Government Regulation Concerning the Labelling and Advertisement of Food, to implement provisions of the Food Law No.18/2012. The draft regulation would prohibit advertising or promotion of milk products for children aged 0–2 years, and the use of claims on foods for children aged 0–3 years; it would ‘severely restrict the infant formula industry’s interactions with health care providers’, and included further stringent requirements for nutrition labelling. The US Government requested Indonesia notify the measure to the WTO TBT Committee, before finalizing the regulation [119].
Japan	Challenging under fill of import quotas in the WTO, and lobbying for enhanced market access	Between 1996 and 2017, WTO members continuously raised concerns about Japan’s under fill of tariff rate quotas (TRQ) on dairy products, including infant formula. In 2015, the US released a press release on the impacts of the concluded negotiations on the Trans-Pacific Partnership Agreement, focusing on agricultural trade with Japan. This said that Japan would establish a transitional country specific quota (CSQ) for US exports of mineral concentrated whey, prepared infant formula, and whey. Between 2013 and 2014, prior to the conclusion of negotiations, dairy industry associations spent US$451,000 lobbying the US Government in relation to the dairy specific aspects of the TPP, mentioning Japan [67]. [Russ K, Baker P, Byrd M, Kang M, Siregar RN, Zahid H, McCoy D: Understanding the global trade and public health regime complex: a case study on breastfeeding and commercial breastmilk substitutes. Forthcoming]
Malaysia	Opposing proposed marketing regulations	In 2014, the Ministry of Health started revising and expanding Malaysia’s existing ‘Code of Ethics’ on the Marketing of Infant Foods and Related Products. This included expanded restrictions on educational, promotional, and marketing practices for infant formula and products for young children, as well as on the use of symbols and trademarked brand names on labels or packaging [116, 119]. The US Government raised questions concerning the evidence used in developing the proposed measure [116].
Philippines	Weakening the country’s Milk Code and implementing regulations	In 2006, the Pharmaceutical & Healthcare Association of the Philippines representing US milk formula manufacturers, and the US Chamber of Commerce (USCC), unsuccessfully appealed to the Supreme Court of the Philippines to rescind new Implementing Rules & Regulations (IRR) of the 1986 Milk Code. The new IRRs would extend products covered to 0–24 months, and ban false health and nutrition claims. The USCC sent a letter to the President of the Philippines, claiming the ‘the country’s reputation as a stable and viable destination for investments is at risk’. Industry lobbyists attempted to transfer the legislative debate in the House of Representatives from the Committee on Health, to the Committee on Trade and Industry, aiming to have the IRRs declared void [120]. In 2012, the Infant and Paediatric Nutrition Association of the Philippines, representing a wider number of corporations, supported a new ‘Milk Monster’ bill that would, among other things, reduce coverage of marketing restrictions to products for ages 0–6 months [120].
Thailand	Preventing the expanded scope of marketing regulations	In 2015, Thailand began drafting a revised version of its ‘Milk Code’, the Marketing Control of Foods for Infants and Young Children and Related Products. This would restrict educational, promotional, and marketing activities, including the use of trademarked brand names, packaging, and symbols, establish stronger penalties for advertising violations, and expand coverage to products for children aged 0–36 months. The US Government made ‘repeated requests’ that Thailand notify this measure to the WTO, which it did in November 2016. In April 2017, the National Legislative Assembly passed revisions to the Milk Code. Although various marketing restrictions, as well as penalties for violations were retained, advertising restrictions for products for ages 12–36 months were removed [119]. In 2017, the US Government reported it was ‘seeking to ensure that Thailand’s final measure ‘takes into account appropriate scientific and technical information in order to avoid any unnecessary restrictions on trade’, and that it had engaged ‘extensively with Thailand’ throughout the period ‘both bilaterally and at the WTO and continues to monitor developments, particularly any potential regulations relating to restrictions on products for young children’ [119].
United States	Lobbying to influence key government programmes and policies	Extensive political activities by Nestlé in the US have been documented [43]. In 2014, Nestlé spent an estimated US$160,000 lobbying in relation to the Special Supplemental Nutrition Program for Women, Infants, and Children (WIC) programme, which provisions free formula for low-income families, and for which companies ‘bid’ to secure preferred provider status in state-level contracts, with bids often at or below cost [43]. In 2015, Mead Johnson called for narrowing the eligibility rules of the WIC programme [121]. Mead Johnson, Nestlé, and dairy company executives, served on the US Dietary Guidelines Advisory Committee [122].
Vietnam	Preventing the expanded scope of marketing regulations	In 2012, the US Embassy in Hanoi unsuccessfully petitioned the Chairman of Vietnam’s National Assembly and other senior ministers, to prevent expanded marketing restrictions for products for ages 0–6 moths to 0–24 months [120]. The letter stated ‘several US companies have contacted the US Embassy regarding their serious concerns about this proposed prohibition … which could have a significant negative impact on their business in Vietnam. We share their concerns’. Further, ‘We have not seen any compelling scientific, legal, or economic argument for changing the current regulatory regime’ [123].

Notes: Lobbying data reported in relation to Canada, China, India and Japan were sourced from the Centre for Responsive Politics

**Fig. 5:**
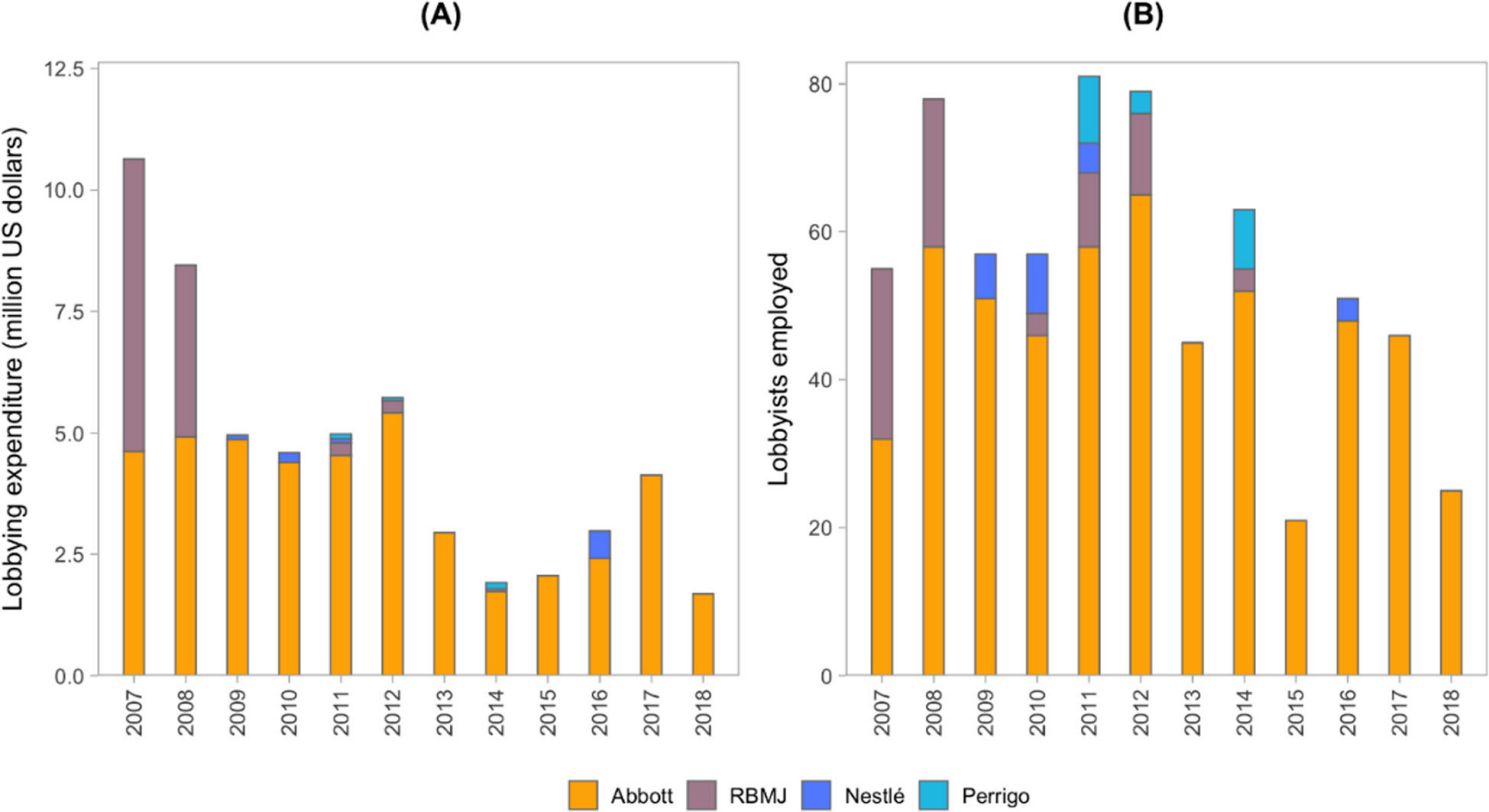
Total BMS-related lobbying expenditure (**A**), and number of lobbyists employed (**B**), by market leading corporations in the United States, 2007–2018. Notes: Data from the Centre for Responsive Politics; data for other corporate entities, including milk formula manufacturers, dairy and other trade associations were excluded due to limited availability, hence these data likely under-represent the true extent of lobbying by the wider industry; RBMJ refers to Mead Johnson, prior to its acquisition by Reckitt Benckiser in 2017

To illustrate this further, and to demonstrate links between lobbying actions in both the national and international arenas, we present US lobbying data. We have already shown how the US has had a disproportionate impact in opposing strong international standards on foods for infants and young children. This influence is understandable given that, between 2007 and 2018, the six US market-leading corporations together spent US$184.2 million in total, on lobbying the US Government [67]. This lobbying was targeted mainly at the Senate, the House of Representatives, and at various times, the Food and Drug Administration, State Department, Office of the US Trade Representative (USTR), and Department of Agriculture (USDA). As Fig. 5 shows, four of these corporations declared lobbying expenditures as specifically related to BMS, totalling US$55.1 million (30% of total lobbying expenditure) or an average of US$5 million per year, with the number of lobbyist they employed fluctuating between 20 and 80 over the time period. Of this BMS-related expenditure, US$43.8 million (79.4%) was attributed to Abbott alone, and US10.1 million (18.4%) to Mead Johnson (all reported prior to its acquisition by Reckitt Benckiser in 2017) [67]. Significant BMS-related lobbying expenditures were also reported by dairy trade associations, food and beverage trade associations, and the Infant Nutrition Council of America [Russ K, Baker P, Byrd M, Kang M, Siregar RN, Zahid H, McCoy D: Understanding the global trade and public health regime complex: a case study on breastfeeding and commercial breastmilk substitutes. Forthcoming].

Of Abbott’s expenditure on BMS-related lobbying, US$20.0 million (45.8%), was dedicated to trade-related concerns. For Mead Johnson, this figure was US$5.8 million (57.2%) [67]. This lobbying was frequently targeted at the State Department and USTR, and to significant effect. As detailed in Table 4, this was reflected in actions taken by the US Government on behalf of the industry to oppose marketing regulations in Hong Kong, Thailand, Malaysia and Indonesia in the WTO, and/or through direct bilateral engagement with governments in national arenas [116, 119]. Lobbying targeted at the USDA was also significant, conceivably because this agency administers the nation’s Special Supplemental Programme for Women, Infants and Children (WIC). Through WIC, the government purchases more than half of all milk formula sold in the US market, and provides ‘nutrition services’ to ~ 1.9 million infants [23, 124, 125]. In 2014, Nestlé alone spent an estimated US$160,000 lobbying in relation to the WIC programme [43].

Political financing is the deployment of financial resources for political gain, in particular payments, gifts or promises, made to elected officials, political parties or government administrators. Big Formula’s corporate policies on political financing vary (Table 5). Danone and RBMJ do not allow it, Nestlé allows it with executive permission, and Abbott (US) allows it. We were unable to source a policy for RFC. However, none of those with policies appear to have prohibited political financing by third parties. Total political financing by the lead US corporations averaged ~US$2 million per year between 1990 and 1999, increasing to US$3 million per year between 2000 and 2012, and have averaged $1 million per year since then. More funds went to candidates for the House of Representatives than for the Senate or White House, and most recipients won their election. In the last 10 years, Abbott’s contributions have steadily grown and now dominate industry political contributions. Political financing also occurs at sub-national levels. Abbott for example, donated $US2.9 million between 2009 and 2019 to state-level candidates and political action committees [126].

### Policy substitution & partnership

The power of Big Formula over first-food systems further resides in the adoption of voluntary self-regulation through corporate policies on responsible marketing, and the acceptance and legitimisation of these private regulatory initiatives by third parties. These policies are summarised in Table 5.

**Table 5:** Corporate policies (voluntary self-regulation) adopted by Big Formula on marketing, lobbying and political financing

Corporation	Nestlé	Danone	RBMJ	Abbott	RFC
Policy name (date of latest version)	Policy and Procedures for the Implementation of the WHO International Code of Marketing of Breast-Milk Substitutes (2017)	Policy for the marketing of breast-milk substitutes; Procedures manual (2018)	Infant & Child Nutrition Pledge; Policy and Procedures on the Marketing of Breast-Milk Substitutes (2018)	Policy on the marketing of infant formula – global policy (2017)	Corporate Policy for the Marketing of Infant Foods; Corporate Standard for the Marketing of Infant Foods (2017)
Year of first corporate policy (revisions)	1982 (1996, 2004, 2010, 2017)	2011 (2012, 2013, 2016, 2018)	2017 (IFM’s policy before this)	2016 (IFM’s policy before this)	–
Compliance with The Code 2018 (2016); ATNI score (#rank)	45% (36%) #2	46% (31%) #1	10% (5%) #5	34% (7%) #3	25% (24%) #4
General compliance statement	Corporate policy, or national regulations, whichever stricter	Corporate policy, or national regulations, whichever stricter	Corporate policy, or national regulations, whichever stricter	Corporate policy, or national regulations, whichever stricter	Corporate policy, or national regulations, whichever stricter
Scope of countries included	‘Higher-risk countries’ only^α^	Worldwide and ‘Higher-risk countries’^α^	‘Higher-risk countries’ only^α^	Worldwide and ‘Higher-risk countries’^β^	Worldwide
Products covered worldwide in corporate policy	–	Standard formula (0-6 m); any other BMS (0-6 m); delivery products; excludes specialised formulas	–	Standard formula (0-6 m); any other BMS, including complementary foods (0-6 m)	Standard, follow-up and special formulas (0-12 m); some products with same brand name / logo
Products also covered in ‘higher-risk’ countries^α^	Standard & follow-up formula (0-12 m); certain specialised formulas; bottles and teats	Follow-up formula (6-12 m); complementary foods & drinks (0-6 m)	Standard & follow-up formula (0-12 m); delivery products; complementary foods (0-6 m); excludes specialised formulas	Standard & follow-up formula (0-12 m); bottles and teats (0-12 m)	–
Corporate third-party auditors listed on website	FTSE4Good since 2011 (PWC audits every 18 months); Bureau Veritas (audits 3 countries / year); ATNI	FTSE4Good since 2016 (PWC audits every 18 months); ATNI; others	ATNI	ATNI	ATNI
Policy on corporate lobbying	Policy on Transparent Interactions with Public Authorities	Global Advocacy Policy 2017	Global Responsible Advocacy Policy	Corporate political participation	–
Policy on political financing	Allowed with executive permission; allowed by third parties	Not allowed; allowed by third parties	Not allowed; allowed by third parties	Allowed (US); allowed by third parties	–

Notes: Data sourced from company websites and reports; α = countries are classed as ‘higher-risk countries’ by the FTSE4Good Breast Milk Substitutes Marketing Criteria when having high rates of mortality (> 10 per 1000) or acute malnutrition (> 2%) in children aged under five; β = for Abbott ‘higher-risk countries’ are defined, although without clarification, by reference to the Global Nutrition Report 2016; Nestlé also has a Code of Interaction with Healthcare Professionals and Institutions for Nestlé Nutrition Business Units, and a Standard for Donations or Low-Cost Supplies for use in Emergencies and for Social Purposes; the others include these in their overarching policies. FTSE4Good commissions Pricewaterhouse Coopers to verify BMS marketing practices against 104 criteria in higher-risk countries. Corporate policies apply to employees of each corporation, and third parties including agents, distributors and other partners. The policies listed in this table only represent corporate policies on BMS; similar policies also exist for several otherenvironmental, social and governance (ESG) issues

The first explicit promotion of self-regulation began in 1975, when ICIFI released a Code of Ethics as a public relations response to the severe public scrutiny Nestlé’s was receiving at the time. The IFM had an industry-wide self-regulatory code, the Rules of Responsible Conduct, until it was disbanded in 2016 [127]. At the country-level, several self-regulatory or co-regulatory codes exist. For example, the Marketing in Australia of Infant Formulas: Manufacturers and Importers Agreement, administered by the Infant Nutrition Council Australia and New Zealand, a trade association representing Big Formula, functions as Australia’s ‘national response’ to The Code. However since 2016, there appears to be no global industry-wide policy. Instead, various policies are adopted across the corporations. Nestlé first adopted such a policy, the Nestlé Charter, in 1982 [128]. Others followed only much later; Danone in 2011, Abbott in 2016, and RBMJ and RFC in 2017. However, these policies fall far short of compliance with The Code, ranging from 46 to 10%, as assessed by the Access to Nutrition Initiative in 2016–18 [129].

In their policies, all state they abide by The Code as implemented by national governments – i.e. as adopted into national laws and regulations – notwithstanding that, through their aforementioned trade associations, they lobby against those very laws and regulations in the first place. The Code applies to all countries, irrespective of their development status. Yet most corporate policies are bifurcated, with stricter standards for ‘higher-risk’ countries with high child malnutrition and mortality rates; the policies of Nestlé and RBMJ only apply to such countries. None apply to products for children beyond 12 months of age, nor address the issue of cross-promotion (see the later section on *marketing strategies*) [129]. At the country-level, subsidiary and affiliate firms often fail to comply with both national regulations and corporate policies [129]. Since 1991, IBFAN [130], and several others [39, 131, 132], have reported extensive Code violations across many countries. These reports are often reviewed and contested by the companies, who consider compliance with local laws and regulations, or their corporate policies only, and not with The Code itself [133].

Big Formula seeks to legitimise their corporate policies, and reinforce their image as socially responsible actors, through ostensibly independent third-party corporate accountability initiatives. Nestlé first initiated this strategy in 1982, when it established the Nestlé Infant Formula Audit Commission (NIFAC), to monitor compliance with its stated commitments in the Nestlé Charter. The over-arching objective was to end Nestlé’s long-running conflict with activists, co-opt more moderate groups, and thereby ‘divide and conquer’. It was to serve as ‘an instrument for damage control and containment’, by re-focusing media attention away from the activists case, and establishing ‘boundaries around the issues that activists might raise, and the manner in which they were addressed’ [134]. Considered an important public relations victory, this contributed to some (although not all) civil society groups ending the Nestlé Boycott in 1984. However, NIFAC was soon considered ‘seriously inadequate’ [98], when continuing country-level violations were reported, and it was disbanded in 1991 [128]. Today, all except Abbott detail internal auditing and compliance processes for their policies, although RFC does so vaguely. Nestlé and Danone also list third-party auditors, of which two are most apparent – the Access to Nutrition Initiative (ATNI), and the FTSE4Good Index as an ethical investment index of the UK company FTSE Russel.

Nestlé was the first to join the FTSE4Good Index in 2011, by meeting its ‘Breast Milk Substitutes Marketing Criteria’ (from hereon ‘Criteria’). In order to join, company policies must ‘align’ with The Code, and comply with national legislation and regulatory requirements. Danone followed in 2016, and then Mead Johnson in 2018, following its acquisition (and subsequent policy revisions) by the existing member Reckitt Benckiser [44]. Other companies, however, have viewed the Criteria as unrealistic ‘because it limits their ability to market’ [135]. The Criteria also falls well short of compliance with The Code. Initially launched in 2001, the Index excluded any company allegedly breaching The Code, and hence no companies were included. The Criteria were revised in 2003, although again no company met the entry requirements. In 2011, it was further revised, this time through a process managed by a small group of industry experts and academics, enabling Nestlé’s entry into the Index. The criteria deals with some issues outside the scope of The Code, for example, by requiring disclosures on corporate lobbying practices and internal compliance systems. However, the new criteria required ‘alignment’ and not compliance with The Code, and as ‘a start’ applied to ‘higher risk’ countries only, which reflected the design of Nestlé’s own policy [135].

Although, some corporations state their policies are not intended as interpretations nor replacements of The Code, Nestlé has represented the FTSE4Good BMS Criteria as an acceptable level of regulation. For instance, in 2017 the company stated ‘More than 35 years after its adoption, only 39 countries have implemented all the recommendations of the WHO Code. To rapidly accelerate progress, all countries that are yet to do so could pass regulations aligned with the minimum standards set by the [Criteria] …’ [136]. However, this would fall far short of full implementation of The Code. Such policies, and third-party auditing reports, are also used directly in public communications to portray Big Formula as compliant. For instance, after ranking second place in the 2018 ATNI assessment, and despite scoring just 45%, a Nestlé press release claimed this reflected it’s ‘commitment to policies, practices and compliance’ with The Code [137]. Earlier, in 2014, the Chairman of Nestlé was quoted as saying ‘We are the only infant formula producer which is part of FTSE4Good. We are being checked and controlled by FTSE4Good. They make their audits in different parts of the world and we have to prove that we are complying with the WHO Code and up to now we can prove that in everything we are’ [138], as quoted in [96].

Despite their violations of The Code, Big Formula’s ‘social license’ to operate under this self-regulatory regime, is further legitimised through partnerships with UN initiatives and agencies. For example, in 2002, Nestlé joined the UN’s Global Compact ‘without challenge’, as arguably the world’s largest self-regulatory initiative, that pledges corporations to abide by ten principles on labour standards, human rights and environmental sustainability [31, 44]. In 2019, Danone partnered with the Food and Agricultural Organization of the United Nations to promote nutrition, food safety and sustainable food systems [139].

### Strategic corporate philanthropy

Another important example of Big Formula’s power is strategic corporate philanthropy, involving the establishment of tax-exempt corporate foundations, as outlined in Table 5, that fund a range of social and environmental initiatives. These further foster an image of corporate social responsibility, and serve directly as a form of promotion.

For example, the Abbott Fund, established in 1951, involves a range of partnerships and funding arrangements ‘to lead change and create new models for health care systems, improve nutrition and address other social needs’, including various child nutrition and micronutrient initiatives across several countries [140]. Since 2012, the Danone Ecosystem Fund has supported the Srikandi Academy, a training institute in Indonesia, through its domestic brand Sari Husada in partnership with the local organization PKPU. By working with key health professional associations, this aims to develop a Ministry of Health endorsed first 1000 days curriculum and toolkits for ‘upskilling’ Indonesia midwives and health workers, with further business coaching, micro-credit and medical equipment made available for establishing practices in rural areas. Through this programme Sari Husada contacts 80,000 midwives each year. As of 2016, it had trained 228 ‘health care cadres’ to engage midwives and spread awareness to mothers, and had sensitised 47,893 people in rural areas to nutrition [141].

Corporate philanthropy also results in direct brand promotion through the donation of surplus products during emergencies, often to humanitarian relief organizations, and well in excess of actual need [142]. In 2000, for example, Wyeth and Nestlé were quoted in a Wall Street Journal article as ready to donate tonnes of free formula for HIV-infected mothers in Sub-Saharan Africa, if asked to by UNICEF. The article framed UNICEF’s refusal to accept these donations as representing a ‘feud against the industry’ and as ‘killing millions of children’ [101]. In 2017, in just one month, Mead Johnson donated enough milk formula for ~ 54,000 child feedings across three US states and territories affected by natural disasters [143].

The Covid-19 pandemic has also been utilised as a marketing opportunity, under the guise of corporate social responsibility. For example, in the Philippines since the beginning of the pandemic (January 2019 to July 2020), there were 291 reported violations of the countries ‘Milk Code’ legislation, compared with 70 in 2019. Of these violations, 235 (81%) were related to donations of BMS products [144]. Covid-19 related marketing violations were reported in Canada, Italy, India, Pakistan, the Philippines and the UK [145]. This included, for example, inferring products boost immunity, associating products with health authorities, offering counselling and support services to parents, and sponsoring health professional ‘educational’ events on Covid-19 and infant and young child feeding [144].

### Co-opting health professionals

The co-option of healthcare professionals in the marketing of their products is a further representation of Big Formula’s power, despite strict provisions in The Code against such practices [33]. A ‘comfortable symbiotic relationship’ between physicians and formula companies has long existed, ever since Henri Nestlé first pioneered the industry’s medicalised marketing techniques in the late nineteenth Century [73], and prescribing formula became a lucrative practice for both. Marketing to health professionals led to formula becoming widely available and used in hospitals throughout the mid-twentieth century in many countries, and both doctors and the public coming to perceive formula as convenient, safe and medically-endorsed, and as associated with modernity and ‘scientific motherhood’ [70–72].

Paediatricians, allergists, nurses, midwives, dietitians, lactation consultants and nutritionists, are among others, trusted sources of infant and young child feeding advice for parents. Medical endorsement bolsters Big Formula’s legitimacy with consumers and policy-makers, and serves as an important form of promotion in itself. A significant proportion of their sales workforce is dedicated to ‘securing the recommendation’. For example, Mead Johnson (now RBMJ) had a global salesforce of 1900 employees in 2010, of which 1350 (71%) were dedicated to health care settings, and the remaining 550 (29%) to pharmacy and supermarket retailers [21]. Techniques used across the industry have included site visits to hospitals, sponsoring new clinical equipment and the design of newly constructed neonatal wards, providing free or low-cost samples for use in maternity discharge packs, providing branded gifts (e.g. lanyards, mugs and pens), paid advertising in journals, various ‘educational interfaces’, and sponsoring professional associations and emerging professional influencers [37, 146]. Some have acted illegally. For example, sales staff at Danone’s subsidiary Dumex, in China, were found to have bribed at least 116 people from across 85 hospitals and health groups, to promote products to parents of newborns [147].

Educational interfaces have included the sponsorship of scientific meetings (e.g. seminars, symposia and conferences), and direct provision of continuing education courses for health workers, delivered on-site and often with meals and refreshments [146], or through extensive online ‘e-learning’ platforms. For example, the latter includes the Nestlé Nutrition Institute, a not-for-profit organization providing 300,000 health professional members worldwide with access to 3000 articles, hundreds of videos, infographics and presentation slides, described as an ‘exclusive accredited e-learning and continuous medical education programs that provide practical guidance on the nutrition of infants and children’ [148, 149]. In some instances, they have partnered with training providers directly. For example, since 2017, Abbott partnered with teaching hospitals in China and Vietnam, to train > 6500 healthcare professionals, providing ‘a model for other hospital pediatric nutrition programs in the region’ [150].

Sponsoring professional associations is widely practiced. A 2019 survey of 114 paediatric association websites found 60% received financial support from BMS companies, ranging from 82% in the Americas to 38% in Africa. Only 16% had published conflict of interest policies, statements or guidelines [151]. This study did not assess sponsorship of other professions, and therefore represents a fraction of total industry engagement. A prominent example, is Big Formula’s long historical relationship with the American Academy of Pediatrics (AAP). As of 2017, excluding payments for advertising and conference exhibits, the AAP was receiving US$3.3 million from four companies every year, accounting for ~ 3% of its annual budget [121]. Big Formula leveraged this relationship through co-branding when, in 2013, US hospitals were reportedly distributing discharge packs of formula samples bearing the AAP logo, and copies of the AAP book on breastfeeding bearing a company brand. This arrangement has since been discontinued [121, 152].

Strategies have also been used to co-opt health professionals in the marketing of specialised formulas [153]. Such formulas are for conditions affecting a small proportion of the infant population, including premature birth, diarrhoea, allergy treatment and prevention [42, 154]. Prescribing behaviours are shaped by research activities, clinical guideline development, medical education and public awareness – all activities Big Formula influences. Industry-driven over-diagnosis of cows-milk protein allergy (CMPA) in particular, has contributed to rapid sales growth in specialised formula sales. In the UK, for example, between 2006 and 2016 specialised formula prescriptions for infants with CMPA increased 500% from 105,029 to 600,000, a rate greatly exceeding any credible change in actual prevalence. This reflected a ~ 700% increase in expenditure by the National Health Service on these products from £8.1 million to >£60 million [153]. The baby food industry, or its marketing consultants, funded the development of at least three clinical guidelines on CMPA, with 81% of all guideline authors reporting a conflict of interest. Furthermore, recommendations made to manage the symptoms as CMA, were found to lack supporting conclusive evidence [153].

Big Formula also works with health professionals to redefine the adaptive food selection behaviours of young children as deviant and abnormal, as conditions that can be eliminated through the use of their products. For example, by collaborating with psychologists, dietitians, and physicians, Abbott, created a new definition for a condition termed ‘feeding difficulties’ [155]. To establish a standard for helping paediatricians accurately identify and manage children with this newly created condition, Abbott funded researchers developed the IMFeD (Identification and Management of Feeding Difficulties for Children) tool in 2011 [156]. Marketing was then employed to fuel awareness of ‘picky eating’ amongst consumers, and to associate this syndrome with poor cognitive and social outcomes for children, thereby appealing to parental anxieties. Advertisements and advice for health professionals focused on prescribing enriched formula milk to children to prevent this ‘state’ [157].

### Capturing the science and knowledge environments

Another key representation of Big Formula’s power over first-foods systems, is scientific capture [158, 159]. To legitimise their products and to support their engagement with health professionals, policy-makers and consumers, Big Formula has acquired vast scientific research capabilities, coordinated through corporate nutrition research divisions, philanthropic foundations and external partners (Table 6). Nestlé, for example, has ‘the world’s largest private nutrition research capability’ with ‘nutritional expertise in every market’ [160]. The Nestlé Nutrition Institute, is not only a ‘continuing education platform’ for health professionals, but also the ‘world’s largest private food and nutrition research organization’, employing ~ 5000 staff across 30 facilities worldwide, and generating ~ 200 peer-reviewed research articles every year [161].

**Table 6:** Big Formula’s corporate nutrition research divisions and philanthropic units, their research capabilities and listed activities

Corporation	Nestlé	Danone	RBMJ	Abbott	RFC
Corporate nutrition research divisions					
Nutrition research division	Nestlé Nutrition Institute (1981) (Not-for-profit association)	Danone Institute International (1991) (Non-profit organizations)	Mead Johnson Pediatric Nutrition Institute	Abbott Nutrition Health Institute (2007)	FrieslandCampina Institute (2001)
Statement of purpose, aim or mission	‘To bring nutrition science to life through the people who live it; connecting a world of healthcare providers, generating discussion and encouraging relevant conversations.’	‘… to promote human health by developing and disseminating knowledge about the links between food and human health, and to highlight the importance of nutrition in human health.’	‘… deliver products with an uncompromising commitment to quality and safety … connects innovative scientific technology and research with cutting-edge manufacturing and quality processes.’	‘… to connect and empower people through science-based nutrition resources to optimize health worldwide.'	‘… shares knowledge and expertise on the nutritional properties of milk and dairy products with nutrition and health professionals, to improve the health and well-being of people worldwide.’
Statement of capability	‘The world’s largest private food and nutrition research organisation, involving around 5000 people located in around 30 R&D facilities worldwide.’	‘… network of 14 Danone Institutes (13 local Institutes and 1 International) … present in 15 countries and gather around 200 experts around the World (nutritionists, pediatricians, gastroenterologists, scientists, sociologists …)’	‘… growing global network of … scientists, research laboratories and facilities … Since 2010 … four new MJPNI research and development technology centers … in the U.S., Mexico, China and Singapore.’	‘Today we support and empower half a million healthcare professionals and the millions of patients they serve.’	‘… approximately 600 … experts across the world’; Institutes in North America, Europe (Netherlands), Asia (Singapore), and Africa; partnerships with dietetic associations, universities and dairy industry communication platforms
Relevant activities listed on website	Digital platform for sharing free resources and content; accredited e-learning & continuing medical education programs; workshops and symposia presentations at congresses; research fellowships in paediatric nutrition and gastroenterology.	Research support through ‘credits, grants, awards, fellowships and scholarships’; including ‘more than 20 different research support programs in 12 countries’; nutrition research prizes; conferences and symposia; publications; nutrition education tools for children.	Research and development; medical education grants provides grant funding for continuing medical education; provides child nutrition advice for parents.	Continuing medical education via online forum, blog site, certificates of training; materials on therapeutic pediatric nutrition; podcasts, webinars and videos on various pediatric nutrition and feeding topics; infographics, scientific articles and briefs.	Stimulating scientific debate and sharing knowledge; engaging governments, NGOs, institutes, scientists and nutrition & health professionals; provides accredited continuing education programs, scientific information, and practical tools.
Corporate philanthropic units					
Philanthropic unit	Nestlé Foundation for the Study of Problems of Nutrition (1966)	Social Innovation Funds	RB Fight for Access Fund (2020)	The Abbott Fund (1951)	–
Status	Philanthropic foundation	Various	Philanthropic foundation	Philanthropic foundation	–
Focus	Initiates and supports research in human nutrition with public-health relevance in LMICs on maternal and child nutrition, including breastfeeding and complementary feeding; nutrient deficiencies and imbalances; interactions between infection and nutrition; and nutrition education and health promotion.	‘We want to support people in adopting healthier and more sustainable eating and drinking practices, and we want to do this in a way that is aligned with our long term commitment to economic success and social progress. ‘	‘To improve access to health, hygiene and nutrition for all. The Fund is, and will, be a demonstration of our Purpose and Fight in action- to protect, heal and nurture in the relentless pursuit of a cleaner, healthier world.’	‘We invest in innovative ideas that expand access to health care, strengthen communities where we live and operate, and promote science and medical education. In partnership with others, we strive to make a lasting impact on people’s lives and encourage others to take positive action.’	–

Notes: Data sourced from company websites and reports

The research generated (and funded) by these platforms promotes a biomedical and nutrient-centric interpretation of infant and young child nutrition, typically focusing on the ‘fortification’ of baby foods (e.g. micronutrient fortified infant cereals), the ‘reformulation’ of products to enhance their nutrient profile (e.g. reduced lactose formulas), or the development of novel product ingredients that ‘functionalise’ their products (e.g. human-milk oligosaccharides) [49, 162]. Such research enables Big Formula to exercise discursive power, and institutionalise certain beliefs and practices in several ways. First, to drive sales by actively portraying their products ‘as close as possible to breastmilk’, and to amplify this message through actual or implied claims about the health and developmental benefits of their products, to both health professionals and consumers (see *marketing strategies*) [163–165]. Second, it shapes wider public perceptions about infant and young child nutrition, by rendering milk formulas as safe, nutritionally adequate and scientific, thereby detracting from the wider health implications of their products [49, 163, 166]. Third, by framing these efforts as part of corporate social responsibility initiatives, this science further legitimises their image as responsible corporate actors and desirable policy partners [49].

Scientific capture also extends into population-level nutrition surveillance research. This not only serves to inform product development across Big Formula’s diverse markets, but also to engage with policy-makers, and through partnerships, with various professional associations, universities and research institutes. For example, RFC has partnered in the South East Asia Nutrition Survey, involving 16,744 children across Malaysia, Indonesia, Thailand and Vietnam, resulting ‘in a better understanding of the diet, health, dietary needs and general dietary patterns of children in Southeast Asia’. Furthermore, the ‘ … findings of the survey have helped local governments and policy makers to develop and implement a scientifically grounded nutrition policy for children in Southeast Asia’ [167]. The purpose of Nestlé’s Feeding Infants and Toddlers Study (FITS) and Kids Nutrition and Health Study (KIDS) is ‘to explore eating patterns, nutrient intakes and food sources of nutrients among infants and children in different countries around the world’. These have involved ‘large-scale cross-sectional surveys’ in Brazil, China, Nigeria, UAE and the US, and studies using national survey data in Australia, China, Mexico, the Philippines and Russia. Since 2002, these studies have collectively generated ~ 90 articles with ~ 100 collaborators [168, 169].

Big Formula also coordinates with other corporate actors, to generate and promote favourable research and knowledge environments. For example, Abbott, RBMJ and Danone, along with various transnational food corporations, are members of the International Life Sciences Institute (ILSI), a corporate-funded organization founded in 1978 by a Coca-Cola Scientific and Regulatory Affairs executive, with the aim of promoting ‘global partnerships for a healthier world’ [170]. Nestlé withdrew its membership in 2020 (this followed the withdrawal of Mars in 2018, citing concerns with ILSI’s ‘advocacy-led studies’) [170]. Despite its claim of being a neutral scientific organization, ILSI members promote industry positions informing health policy responses across many countries and nutrition issues [171–173]. Through its Washington D.C. headquarters, and eighteen branches, its member activities are coordinated across all regions under a ‘One-ILSI’ strategy [170]. As an example, in 2008–2009 members established the South East Asia Region’s Technical Committee and Expert Panel on Maternal, Infant and Young Child Nutrition. During this period ‘in collaboration with seven regional health and research agencies, a total of six Expert Consultations, 11 Seminars and Workshops [were] held in the region with 13 scientific papers published in peer-reviewed journals’. Demonstrating its reach and influence, ‘About 1,000 nutrition, public health and pediatric professionals from government agencies, health and research institutions, NGOs and the private sector attended the meetings’ [174].

### Marketing strategies

As they accumulate greater resources, Big Formula can employ world-leading advertising, branding and public relations agencies, to implement more intensive and sophisticated forms of marketing [4]. The term ‘marketing’ includes a set of corporate strategies – the so-called ‘marketing mix’ – including product design, pricing, advertising and promotion, retail placement and public relations strategies, among others. Such marketing effectively undermines breastfeeding; exposure is associated with reduced initiation, exclusivity and duration in all country contexts [13–15]. Within countries, marketing exposure appears to ‘cascade’, concentrating initially in first-tier cities with higher income consumers, before becoming more prominent in peri-urban areas, lower-tier cities and towns, as has occurred in China [175].

Big Formula are among the world’s most recognised and valuable brands, and largest advertising spenders. Nestlé, for example, is among the world’s most recognised household names, with its brand valued at $US12.6 billion in 2019, ranked 50th among global brands. The company spent an estimated US$9.9 billion on ‘consumer facing’ advertising in 2016, the third highest spender worldwide [176]. Although we could not source data on Big Formula’s marketing expenditures specifically, we used a conservative estimate of ~ 3–10% of sales [42], to estimate a global spend of between US$1.68–5.56 billion in 2019. These figures far outweigh any expenditure on breastfeeding promotion by governments, international organizations and global health donors [146]. Some national marketing data are available, and indicate a significant increase in expenditure on milk formulas for older infants and young children, relative to infant formula. For example, in 2015, three companies Nestlé, Abbott and Mead Johnson spent US$9.75 million on advertising infant formula in the US market, and US$16.83 million advertising toddler milk [41].

Big Formula contract agencies to develop and execute their strategies. The globalization and consolidation of the advertising sector since the 1980s has been well described. A small number of ‘global communications groups’ based in the US, Europe and Japan, control most of the world market through networks of subsidiary agencies. These have typically followed their corporate clients into new markets, hence globalizing alongside them, and providing coordinated services across diverse markets [28, 86]. This sector features significantly in Big Formula’s global influence network (Fig. 3). The entry of a corporation into a new market can change the intensity and forms of marketing by the industry as a whole. For example, prior to the mid-1980s, the three largest US manufacturers practiced a voluntary ban on advertising in agreement with the American Academy of Pediatrics (AAP), and as pharmaceutical companies, they could rely upon their extensive sales networks to health care providers. With no such network in place, Nestlé decided to ignore this agreement when it entered the market in 1985, and began direct-to-consumer advertising in 1988 [71]. In 1993, it filed a lawsuit against the US companies and the AAP, claiming the advertising ban was a barrier to trade in violation of US competition law [27].

The first key pillar of Big Formula’s marketing strategy is health professional co-optation, which we have already described (see *co-opting health professionals*). Direct-to-consumer advertising is the second pillar. Traditional advertising channels include parenting magazines, television and in-store retail displays [40, 146]. As digital technologies expand worldwide, parents and especially mothers, are increasingly engaged through social media platforms, parenting forums, mobile apps, e-commerce sites, reward programmes and sponsored parenting blogs [146, 164, 177, 178]. Celebrities and other influencers are employed to promote products on social media, and to host events on- and offline [178, 179]. Health professionals and other experts host question and answer sessions and webinars on infant feeding and other lifestyle topics on social media [179, 180]. Advertisements often contain links to clubs, carelines and promotion lines, where women are encouraged to engage with industry representatives and health professionals in a one- to-one conversation [181].

‘Big Data’ analytics platforms enable sophisticated forms of market segmentation and targeting, as personal data can be collected and then used to generate tailored, and even personalised, advertisements to parents differentiated by, for example, income, parenting stage and lifestyle [19, 41, 42, 182]. Messages often portray milk formula as a symbol of modernity, as equivalent with or superior to breast milk, and formula-feeding as extensively practised, and as a desirable lifestyle choice [41, 146, 183]. Appeals are made to the emotional and psychological aspects of parenting (e.g. ‘when mothers milk fails’ and ‘freedom from judgement’), tensions between formula-feeding and breastfeeding parents (e.g. Abbott’s The Sisterhood of Motherhood campaign), and parental aspirations (e.g. child learning ability, paternal bonding, and minimising parent-child conflict) [41, 183].

The third key pillar of Big Formula’s market strategy, is product innovation. In a process of market segmentation, the creation of entirely new product categories is used to not only to generate new sales growth, but also to circumvent marketing regulation. By the late 1970s, 200 infant formula products and 50 brands were reportedly available across 100 countries [42]. However, prior to The Code infant formula was the main product category, promoted ‘from birth’ without upper-age limit. From 1984 onwards, just as governments were beginning to implement The Code, the marketing of follow-up formulas and toddler-milks markedly intensified, and soon became widely available. This was described by one industry report as the renaming of products ‘primarily to avoid regulation and restrictions on advertising’ applying to the first 6 months only [21]. By using nearly identical branding and labelling across their entire product range, Big Formula have ‘cross-promoted’ their products, including infant formula in countries where legislation prohibits this [42, 165, 166, 184]. This strategy of cross-promotion also extends from ‘womb-to-tomb’, through branded milk formula products for all life-stages, including infants and young children, but also for pregnant and lactating mothers, older children and adolescents, and the elderly (i.e. grandparents) who are also often involved in making feeding decisions [42].

Finally, Big Formula’s aforementioned scientific capabilities are also used to support ‘nutritional positioning’, a marketing technique involving the development of products with novel ingredients and implied or direct health claims, often on premium or specialised products that sell for markedly higher prices [42, 163–165]. Ingredients once found only in specialised formulas have been increasingly added to nearly all products. Many claims have no publically available evidence, or only poor-quality evidence, to support them [185, 186]. The evidence that does exist, often cites studies directly sponsored by Big Formula [187]. Claims made on product labels include inter alia those relating to brain, eye and immune system development, reduced allergies, and to specific outcomes linked with normal infant behaviours, including sleeplessness, fussiness and regurgitation [188, 189]. These claims are supported by the addition of functional ingredients claimed to mimic breastmilk (e.g. human-milk oligosaccharides, essential fatty acids, and probiotics), or reformulated ingredients (e.g. reduced lactose) [186, 188].

When combined, these techniques powerfully influence social norms and beliefs about what feeding practices are considered normal, acceptable and socially desirable [42, 146]. For example, one recent US survey found 52% of caregivers agreed with the statement that infant formula can be better for babies’ digestion and brain development than breastmilk; 62% that it can provide nutrition not present in breastmilk [161].

## Discussion

Here we consider how this study advances our understanding of corporate power and the commercial determinants of maternal, newborn and child health. Our results show that milk formula is a phenomenal commercial success – a global market worth ~US1.5 billion in 1978, is today worth US$55.6 billion, representing a 36-fold increase over a 40-year period. How then, in-spite of The Code, and the ever-growing evidence on the harms of formula-feeding, has the industry managed to sustain this remarkable growth? In our view, this historical expansion reflects the core underlying driver of capitalism itself – the pursuit of profit and capital accumulation – and through this pursuit, the subjugation of the mother-child breastfeeding dyad to corporate power, and the transformation of first-foods systems to promote and sustain high levels of milk formula consumption.

### Commodifying the mother-child feeding dyad

The global rise of milk formula, by necessity, requires the transformation of infant and young child feeding into an object of trade. This has involved the replacement of breastfeeding, as the biological norm and first-food supply chain, with formula-feeding and commercial supply chains, across an ever-widening population of children. Our results show this process has occurred along two main historical axes.

First, through the expanding geographical reach of milk formula marketing. Across countries, this has involved a handful of corporations originating mainly in the advanced capitalist economies of Europe and the United States, expanding initially in the Global North alongside historic declines in breastfeeding, and then as those markets have matured and stagnated, expanding intensively into countries throughout the Global South, with much higher, although in some cases now greatly diminished, breastfeeding rates. Within countries, markets appear to expand first among urban elites in major urban centres, before spreading more widely into lower-tier cities and towns, and among poorer population groups. The growth of modern retail outlets, the increasing hospitalisation and medicalization of birth, and the emergence of large-scale grey market trade, have created key distribution channels for making milk formula widely available.

Second, through widening the boundaries of mother-child populations subject to commodification. Product ranges have expanded from largely a single infant formula category prior to the 1980s, to include follow-up formulas, toddler milks and an array of specialised formulas, along with milks for pregnant and lactating mothers and so on. Industry-driven over-diagnosis, the invention of new feeding ‘conditions’, and the expanded consumption of specialised milks that results from this, is consistent with a strategy of ‘disease-mongering’, intended to widen the ‘boundaries of illness and grow the markets for those who sell and deliver treatments’ [190]. This combined process of market segmentation and mongering has greatly expanded the age range of children consuming formula, and indeed the consumption of branded milk drinks across the entire life-course [37].

### The power of marketing

As markets have expanded in both geographical reach and population scope, Big Formula have used sophisticated marketing techniques to influence choice, drive consumption, and normalise formula-feeding across diverse country contexts. Arguably, as this normalisation occurs, corporations become key (if not the main) custodians of knowledge and education about infant and young child feeding. This power is enabled through massive expenditures on branding, advertising and sales promotion, bolstered by the creative expertise of global advertising agencies. Corporate science conducted by the corporations themselves, and through engagement with external scientists and organizations, is used to support and amplify this marketing. Broadly consistent with earlier studies [37, 41, 146, 191], we identify three key pillars of Big Formula’s marketing strategies.

First, is health professional co-option to ‘secure the recommendation’, distribute formula through health systems, and legitimise milk formula products as safe, scientific and medically-endorsed. Notably, we identify the significant structural power Big Formula holds over the training of health professionals in paediatric nutrition, and infant and young child feeding more broadly, through wide-reaching sponsorship of professional associations, and through the direct provision of professional education, including through large-scale e-learning platforms. The power of this ‘corporate education’ is amplified, when breastfeeding is often excluded from the curricula of healthcare professional training providers – a serious problem recognised worldwide [192–194].

Second, direct-to-consumer advertising occurs through mass- and digital media. Messaging promotes formula as scientific, medically-endorsed, and as equivalent with, or as superior to breast milk, along with appeals to parental anxieties, aspirations and lifestyles [41, 146, 183]. Global growth in digital technologies, including social media and smart phones, has enabled sophisticated new data collection and analytical techniques, to personalise and deploy targeted advertisements. The power of digital marketing should not be underestimated, and likely represents a key driver of the recent surge in global milk formula sales, as well as a key challenge for national regulators [181]. The Committee on the Rights of the Child recognises the potential for digital advertising and marketing to violate or abuse child’s rights, and calls on governments to prohibit by law, the profiling or targeting of children [195].

Third, are product innovations. The segmentation and extension of milk formula product ranges not only widens the boundaries of markets and cultivates new demand, as described earlier; it also enables the cross-promotion of products across the entire branded range, and the circumvention of marketing regulations applying to infant formula only [42, 46, 184]. Nutritional positioning, involving the development of novel products with reformulated and functional ingredients, and the use of direct or implied health claims, reinforces an image of milk formula as an optimal form of early-life nutrition [42, 163–165]. These are marketing techniques requiring much greater scrutiny by regulators.

### The power to market

However, the capacity to deploy the marketing techniques described above is only possible because of the large investments the baby food industry makes in fostering policy, regulatory and knowledge environments conducive to such marketing in the first place. We find extensive evidence of the industry’s political practices, coordinated on a global scale, to achieve this. When considered together, these demonstrate two faces of corporate power – a more hidden, covert one, involving strategies to constrain critical discourse, co-opt opponents, and curtail regulatory threats; and a more visible public-facing one, to foster an image of corporate social responsibility, and maintain their ‘social license’ to operate [28, 31, 44]. These practices are broadly consistent with those identified in studies on the tobacco, alcohol and ultra-processed food industries (e.g. [196–198]), and how neo-liberal economic globalization has markedly strengthened the power of these industries, to grow and sustain their markets, e.g. [58, 85, 196–199]).

First, the baby food industry employs a large global influence network, comprising many trade associations, addressing diverse corporate issues and regulatory threats. Lobbying practices by this network are often less visible or invisible to outside scrutiny, and appear to be coordinated on a global-scale. Such practices are strongly incoherent with Big Formula’s corporate social responsibility initiatives. The corporations state they abide by their own policies, and by the laws in the countries in which they operate; but then belong to trade associations that lobby against the adoption of those very same laws. Hence they foster an image of responsible conduct, but allow third-parties to undermine breastfeeding on their behalf, without tarnishing their reputation. This might be referred to as a strategy of ‘political distancing’.

Infant and nutrition trade associations, which are largely funded by Big Formula, appear to be ‘core’ to this network. Others, including branding and advertising associations, also potentially represent key impediments to worldwide implementation of The Code, although this requires further investigation. In the US we found that lobbying expenditures declared by Big Formula are extensive, and highly targeted at various government agencies, yet have declined. This may reflect actual declines, or a reduction in corporate disclosure, or reductions in the cost of lobbying. It is also possible that lobbying activities have become increasingly outsourced.

Second, Big Formula’s corporate policies on responsible marketing are consistent with a strategy of ‘policy substitution’, one that aims to pre-empt, delay and/or replace regulation by the state. Although The Code stipulates manufacturers and distributors should monitor ‘their marketing practices according to the principles and aim of [The Code], and … ensure that their conduct at every level conforms to them’ [32], their self-regulatory initiatives establish a global response far short of compliance with The Code. At the same time, the legitimisation of this self-regulation through third party corporate accountability initiatives, helps to portray Big Formula as responsible corporate citizens, while simultaneously deflecting blame, and co-opting moderate opponents [48, 200]. This image of responsible conduct, is further bolstered by strategic corporate philanthropy. This not only includes social and environmental initiatives, but also what we call ‘crisis marketing’, including large-scale product donations and others marketing techniques to exploit emergencies, or crises, like Covid-19.

Third, the governments of large dairy-producing and exporting nations have consistently acted on the industry’s behalf to influence standard-setting processes in international policy fora, and taken bilateral actions to weaken implementation of The Code by other governments through ‘economic diplomacy’. At the international level, three multi-lateral institutions have been the focus of this influence – the WHO, CAC, and the WTO, that together establish and (in the case of WTO) enforce standards, on the marketing, safety, labelling, composition and trade of BMS [Russ K, Baker P, Byrd M, Kang M, Siregar RN, Zahid H, McCoy D: Understanding the global trade and public health regime complex: a case study on breastfeeding and commercial breastmilk substitutes. Forthcoming]. In CAC standard-setting processes, the distinction between ‘member state delegate’ and ‘industry observer’ is somewhat blurred, and very often industry representatives *are* the member state. Such standard-setting processes therefore appear to be strongly ‘captured’, suggesting the need for much greater scrutiny of how industry participates at Codex, and how to bolster representation by public-interest civil society groups.

Many governments have been challenged in the WTO when attempting to implement The Code into national law, and in several cases this has resulted in weakening of the scope and strength of regulation. It is also likely this has a much wider ‘chilling’ effect on other governments, who after having observed these challenges, re-consider their own commitment to regulate BMS [58]. The US Government in particular, has been a remarkable force in weakening the legal status and provisions of The Code at the WHA, and its implementation by other governments into national law. This appears to be linked with a small number of US corporations and trade associations, who have consistently lobbied US Government agencies on BMS-related trade issues. This represents a major area of policy incoherence for the US Government, given it is the largest contributor of overseas development assistance for breastfeeding [201].

Finally, the baby food industry is currently undergoing terminal consolidation with markets now moderately to strongly oligopolistic. With consolidation, Big Formula have accumulated vast material resources, and become structurally important within various national economies as suppliers of jobs, investments and export earnings [2, 202]. As a result, governments have directly supported the industry’s growth through lax domestic regulations and other policy measures. For example, the US, Canada, New Zealand and Australia, all major milk formula and dairy producing nations, have yet to adopt any provisions of The Code into national law.

The US Government effectively subsidises the industry, by purchasing half of all formula sold through its WIC Program [124, 125]. In China, the Government provides direct support through a whole-of-government policy, including tax cuts, approvals for industry consolidation, and finance to support foreign acquisitions [203]. Food regulatory environments in many countries are permissive of health and nutrition claims on milk formula products, with low-evidential requirements, thereby enabling the marketing technique of ‘nutritional positioning’. This calls into question whether existing regulatory systems are fit-for-purpose in meeting public health objectives [185, 204].

### Limitations and strengths

This study has several limitations and gaps, which require investigation. First, there is a strong US-bias in our data. This is because US corporate lobbying disclosure and transparency laws have enabled us to access relevant data, whereas we were unable to source useful comparable data from the EU or elsewhere. Second, we have not examined the financiers behind the baby food industry. Others have reported on how the financialization of the global economy, involving the emergence of a liberal financial regime, has helped facilitate the global value chain integration of transnational corporations, and provided access to finance and risk management techniques for accelerated global expansion [19, 205]. Furthermore, we have given only cursory consideration to the evolving yet important role of the global trade and investment regime, in facilitating milk formula market expansion.

Third, we have not engaged with feminist economic perspectives to understand the core economic drivers of first-foods systems, including the underlying (dis) incentives that compel governments and other actors to support the growth of milk formula markets over breastfeeding [5]. For example, commercial milk formulas are represented by industry groups and governments as high-value export commodities that support ‘jobs and growth’, and get ‘counted’ in national accounting systems and gross domestic product (GDP), whereas the immense societal value of breastfeeding and other forms of informal care work provided by mothers, does not. Fourth, we have not considered the role of manufacturers and distributors of bottles, teats and other feeding apparatuses in shaping first-foods systems, although these products are also covered by The Code.

Fifth, we have not considered the overall distributive effects of the industry. Yet, not breastfeeding generates global economic losses of US$341.3 billion annually, resulting from higher health care costs, premature mortality and lost productivity [9]. The production of ingredients and the manufacturing and consumption of milk formula generates significant environmental harms, including water pollution, greenhouse gas emissions and packaging waste [206]. Hence the power of Big Formula to expand markets, and accumulate profits, extends at least partly from its power to externalise the social, economic and environmental costs of production. These wider distributive effects of the industry, and its contribution to sustainable economic development, requires much greater scrutiny.

Finally, we have not directly examined how corporate power influences feeding practices by the mother-child feeding dyad itself. Research on ultra-processed foods suggests that market expansion is partly enabled by a skills transition, as consumers appropriate new ‘techno-skills’ required to source, prepare and consume such foods [19]. The same is likely true for milk formulas – the skill of breastfeeding, which has traditionally been transferred inter-generationally through women-to-women and kin-based relationships [6], is displaced by new skills required for artificial feeding, informed by ‘commercial education’ and marketing [28].

The main strength of this synthesis is that we describe how corporate power works to shape first-foods systems in their entirety, rather than just focusing on certain features or sub-systems. In doing so, we have provided a macroscopic understanding of how corporate power works, across an entire system, historically and in global context. We were able to do this by using a multi-disciplinary approach and theoretical framework to guide the study, thereby increasingly the likelihood we captured the main market and political practices of the industry. This was also enabled by drawing from diverse data sources, including extant literature, but also new empirical data where the literature was sparse or missing altogether, as well as the perspectives of our large multi-disciplinary research team.

## Conclusion

Milk formula, first invented in Europe in the 1860s as a specialised product for the small proportion of the infant population unable to breastfeed, has become a mass-produced consumer good, available nearly everywhere. Our findings show how Big Formula, and the wider industry, have used diverse market and political practices to powerfully shape first-foods systems in ways that drive the expansion of milk formula markets on a global scale. This has resulted in an unprecedented increase in the number of children consuming milk formula worldwide, and represents a significant impediment to global progress on breastfeeding. The historical expansion of the industry has occurred across two main axes – first, through the expanding geographical reach of milk formula markets across and within countries; and second, the broadening of milk formula product ranges, and hence the scope of mother-child populations subject to commodification.

The industry has harnessed the power of marketing to grow and sustain high levels of milk formula consumption, including most recently through the use of sophisticated digital marketing techniques. Despite The Code, and other public health actions to curtail the industry’s marketing practices, milk formula markets have massively expanded. This power of marketing is only made possible, because of the large investments made by the industry in fostering favourable policy, regulatory and knowledge environments that enable and sustain such marketing in the first place. Responding to this challenge, and accelerating global progress on breastfeeding, will require the strengthening of worldwide actions to constrain *the power of marketing*, including through the use of law and government intervention. However, it will also require actions to constrain the baby food industry's *power to market*, by targeting the political practices of the corporations, and their global network of trade associations and other corporate-funded influence organizations. This presents a formidable challenge, and suggests that new modalities of public health action are urgently needed.

## Supplementary Information

**Additional file 1: Figure S1.** Global dry milk powder production and trade dynamics in 2005 (top) and 2014 (bottom) – circles represent country production values (tonnes), and lines the value and direction of trade (US$). Notes: To simplify the figure, only countries with trade flow (export) values >US$10 million were represented; dry milk powder production values (tonnes) were sourced from FAOSTAT. Trade data were soured from UN Comtrade. **Table S1.** List of organizations in the baby food industries’ global influence network. Notes: * formerly the Grocery Manufacturers Association.

## Data Availability

All data generated or analysed during this study are included or cited in this published article and its supplementary information files. The information contained in this manuscript has been obtained from sources believed to be reliable. However, any potential interpretation of the findings as making an allegation against a specific named company or companies would be incorrect and misleading.
