# Supplementary figures and images for: Globalization, first-foods systems transformations and corporate power: a synthesis of literature and data on the market and political practices of the transnational baby food industry
Source: Global Health. 2021 May 21;17:58. doi: 10.1186/s12992-021-00708-1 (PMC8139375; doi:10.1186/s12992-021-00708-1)

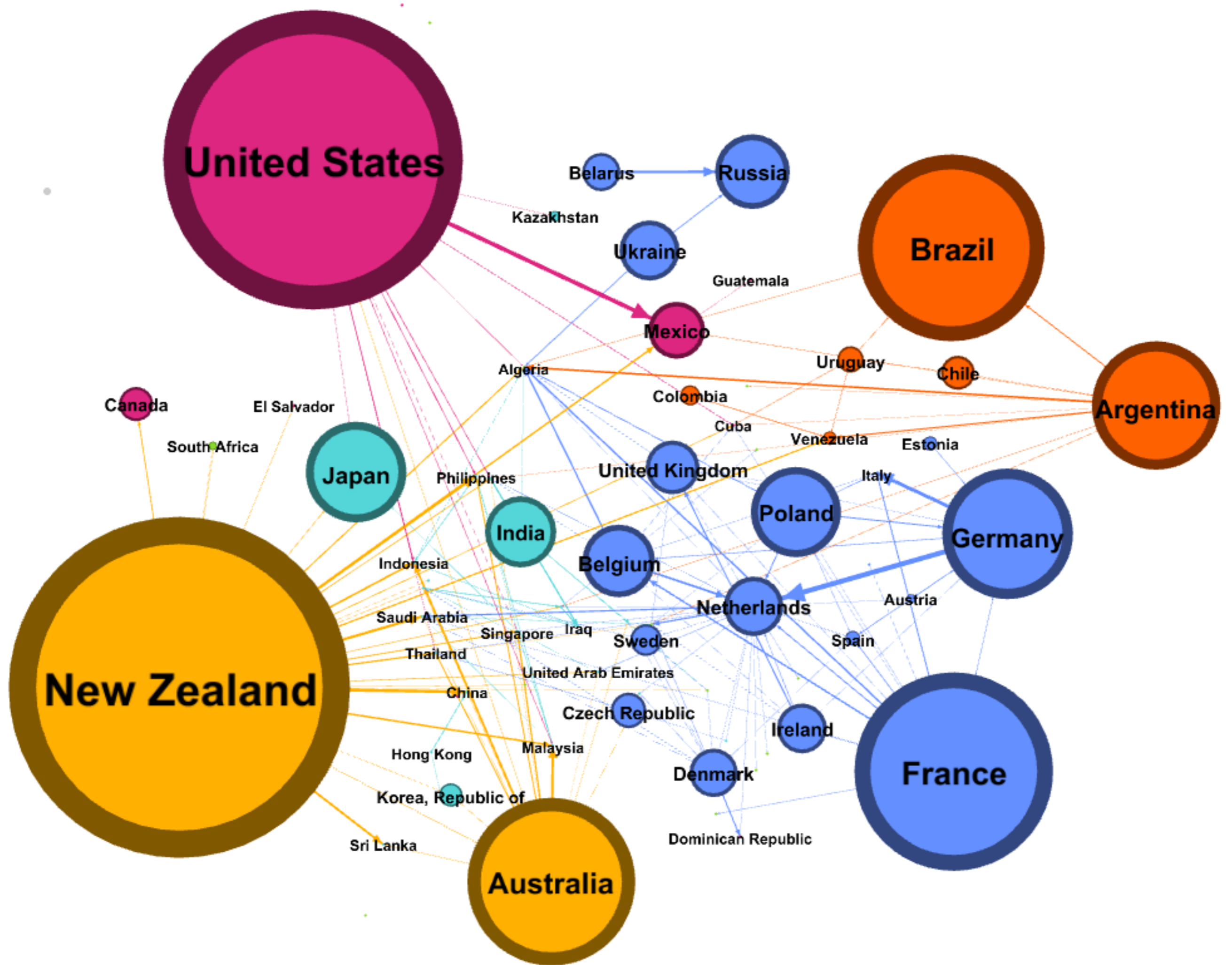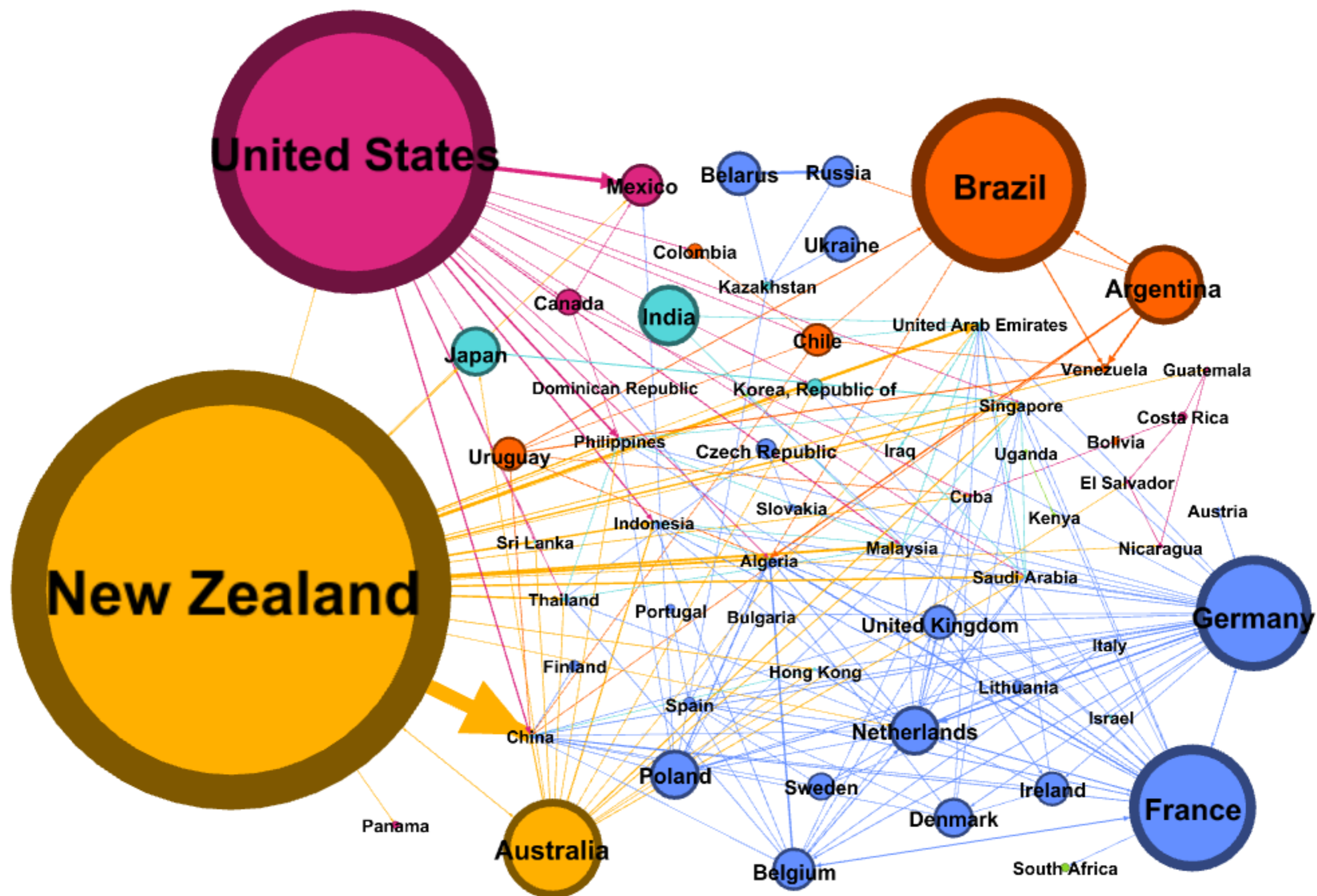

Supplement: Supplementary file 1 — Additional file 1: Figure S1. Global dry milk powder production and trade dynamics in 2005 (top) and 2014 (bottom) – circles represent country production values (tonnes), and lines the value and direction of trade (US$). Notes: To simplify the figure, only countries with trade flow (export) values >US$10 million were represented; dry milk powder production values (tonnes) were sourced from FAOSTAT. Trade data were soured from UN Comtrade. Table S1. List of organizations in the baby food industries’ global influence network. Notes: * formerly the Grocery Manufacturers Association. [file 12992_2021_708_MOESM1_ESM.pdf]
